# CO_2_ Mineralization by MgO Nanocubes in Nanometric Water Films

**DOI:** 10.1021/acsami.3c10590

**Published:** 2023-09-14

**Authors:** N. Tan Luong, Noémie Veyret, Jean-François Boily

**Affiliations:** Department of Chemistry, Umeå University, SE 901 87 Umeå, Sweden

**Keywords:** air moisture, CO_2_, mineralization, magnesium oxide, magnesium carbonate, water films, nanomaterials

## Abstract

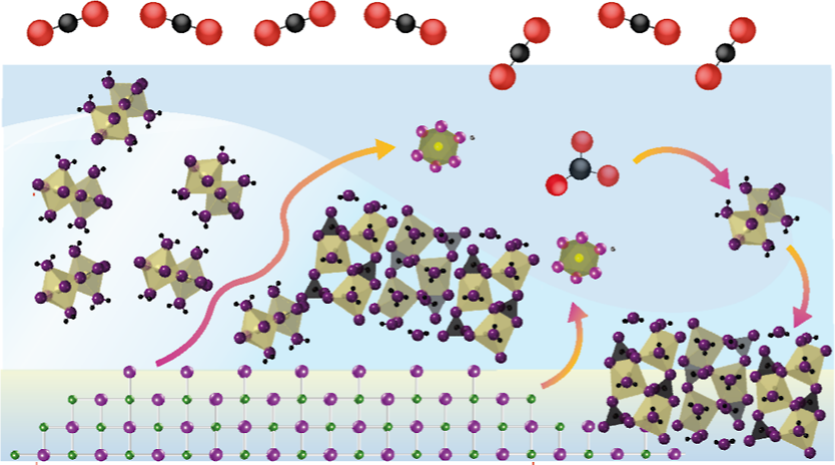

Water films formed by the adhesion and condensation of air moisture on minerals can trigger the formation of secondary minerals of great importance to nature and technology. Magnesium carbonate growth on Mg-bearing minerals is not only of great interest for CO_2_ capture under enhanced weathering scenarios but is also a prime system for advancing key ideas on mineral formation under nanoconfinement. To help advance ideas on water film-mediated CO_2_ capture, we tracked the growth of amorphous magnesium carbonate (AMC) on MgO nanocubes exposed to moist CO_2_ gas. AMC was identified by its characteristic vibrational spectral signature and by its lack of long-range structure by X-ray diffraction. We find that AMC (MgCO_3_·2.3–2.5H_2_O) grew in sub-monolayer (ML) to 4 ML thick water films, with formation rates and yields scaling with humidity. AMC growth was however slowed down as AMC nanocoatings blocked water films access to the reactive MgO core. Films could however be partially dissolved by exposure to thicker water films, driving AMC growth for several more hours until nanocoatings blocked the reactions again. These findings shed new light on a potentially important bottleneck for the efficient mineralization of CO_2_ using MgO-bearing products. Notably, this study shows how variations in the air humidity affect CO_2_ capture by controlling water film coverages on reactive minerals. This process is also of great interest in the study of mineral growth in nanometrically thick water films.

## 1 Introduction

Water films formed by the adhesion and condensation of air moisture on nanominerals are reactive solvation nanoenvironments that can drive important phase transformation reactions. Gas and mineral dissolution reactions can be key drivers for nanocoating growth or complete transformation of host particles into secondary minerals. Magnesium carbonate growth on Mg-bearing minerals is of particular interest for air capture technologies^1−6^ and notably those making using of enhanced^4−11^ mineral weathering. The scientific basis for this form of CO_2_ mineralization stems from earlier work of Seifritz^7^ and Lackner,^8^ where reactions with ground rocks could be enhanced to remove atmospheric CO_2_ on human timescales. This and related technologies require a deepened understanding of the mechanisms of mineral transformations as materials are exposed to moist (ambient to pressurized) CO_2_-bearing gases. Advancing this knowledge has the added benefit of providing new opportunities for tracking mineral growth phenomena under the confines of molecularly thick water films, which are the key mediating solvents for CO_2_ capture.

**Figure 1:**
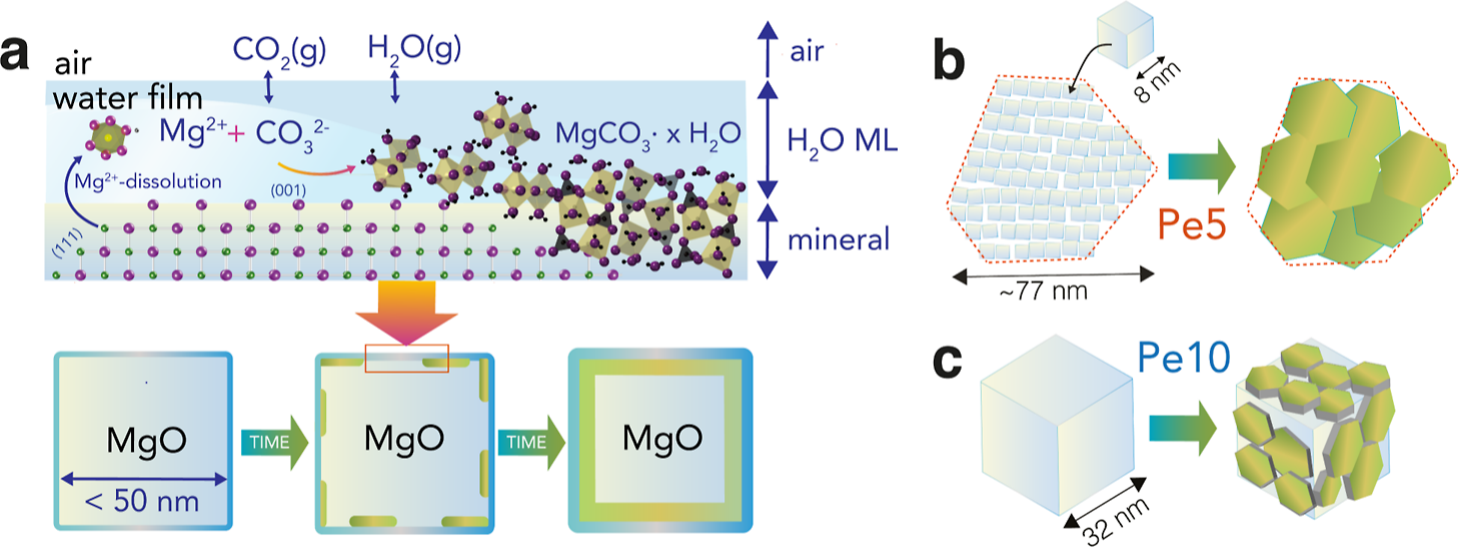
Schematic representation of water film-driven growth of magnesium carbonate at periclase nanocube surfaces. (a) Depiction of concurrent MgO and CO_2_(g) into water films, Mg^2+^–CO_3_^2–^ complexation, and precipitation to magnesium carbonate nanocoatings (green) on single (*e.g.*, <50 nm wide) nanocubes, leaving an unreacted core. (b, c) Contrasting secondary mineral growth (b) limited to the confines of aggregated Pe5 nanocubes [produced by Mg(OH)_2_ dehydroxylation at 500 °C] and (c) expanding from a collection of monodispersed Pe10 nanocubes [produced by Mg(OH)_2_ dehydroxylation at 1000 °C], as we described in a previous study.^12^

To better understand CO_2_ mineralization reactions in water films, we monitored magnesium carbonate precipitation reactions in MgO nanoparticles exposed to water vapor. MgO is not only an important building block of ultramafic mine wastes currently considered in CO_2_ mineralization technologies^1,2^ but can also be synthesized for deepened studies aimed at advancing knowledge of these and related reactions.^13−18^ Exposing MgO nanocubes to ambient water vapor grows water films of only a few monolayers (MLs) which, in the absence of CO_2_, produce brucite nanosheets (MgO + H_2_O → Mg(OH)_2_).^12,16,19,20^ Dissolving CO_2_ in water films however promotes CO_2_ mineralization by securing a flux of (bi)carbonate ions reacting with Mg^2+^ ions directly at MgO surfaces or dissolved in the water films (Figure 1a). The solvent-driven like carbonation reaction in thin water films can be related to other CO_2_ sequestration reactions in aqueous solutions. Such reactions under ambient conditions tend to produce hydrated amorphous magnesium carbonate (AMC; MgCO_3_**·**0.5–3H_2_O)^21−24^ that subsequently transforms to hydroxycarbonates [Mg_5_(CO_3_)_4_(OH)_2_·∼4–8H_2_O], nesquehonite (MgCO_3_**·**3H_2_O or MgHCO_3_**·**OH**·**2H_2_O), or dypingite^25^ [Mg_5_(CO_3_)_4_(OH)_2_**·**5H_2_O], rather than crystalline magnesite (MgCO_3_). This is understood by the high affinity^26−29^ of first shell water molecules to the Mg^2+^ ions which require high activation energies to produce anhydrous MgCO_3_.^30^ This energy barrier can however be overcome by reactions in solutions of low water activity or at extremely high pressures and temperatures.^29−34^ Still, carbonate products often contain a mixture of various hydrated intermediate phases, which are more kinetically favored, over a period of days to months.^32^ For instance, high temperatures can convert AMC to hydromagnesite [Mg_5_(CO_3_)_4_(OH)_2_·4H_2_O] without substantially altering local structure.^35^ Alternatively, vigorous stirring of AMC suspensions can rework the 3D hydrogen bonding environment of AMC units, thereby inducing alternative energetically favorable pathways for phase conversion.^36^ This could align with recent work^23^ showing that the local structure of AMC can be related to those of nesquehonite and to hydromagnesite.

Magnesium carbonate growth in unstirred, passive, nanosolvation environments of thin films could however lead to contrasting reaction products. From previous work,^21−24,29−33,37−40^ we expect that low temperature and low pressure conversion reactions, which can be needed for low-cost passive CO_2_ uptake,^11^ would lead to contrasting reaction products to those that have thus far been chiefly characterized at high temperatures and pressures.^41^ Also, considering that water film thickness responds directly to variations in atmospheric humidity and temperature,^42−44^ tracking magnesium of carbonate growth under experimentally controlled solvation environments is needed to understand the impact of water film loadings on growth yield and rates.

To evaluate the uncertainties that these potential shortcomings pose on CO_2_ capture, we tracked mineralization reactions from MgO nanocubes over a range of atmospheric moisture and temperatures. These reactions were resolved in synthetic MgO nanocubes of contrasting aggregation modes and particle sizes.^13−16^ In recent work,^12,20^ we showed that ∼8 nm wide MgO (Pe5) nanocubes (Figure 1b) were sufficiently small to completely convert to brucite when exposed to CO_2_-free water films. Here, brucite grew within two-dimensional hexagonal casings of aggregated Pe5 nanocubes.^45^ In contrast, brucite nanocoatings grown on ∼32 nm wide MgO (Pe10) nanocubes (Figure 1c) blocked the reactions, leaving an unreacted MgO core. In this study, we build upon these findings to resolve carbonation reactions on these particles. We show that AMC nanocoatings prevent full carbonation, even of the otherwise highly reactive ∼8 nm wide MgO nanocubes. These findings shed new light on a potentially important bottleneck for the efficient mineralization of CO_2_ by MgO-bearing products, as well as by other related minerals that are potential candidates for low-cost CO_2_ capture.^1^

## 2 Methods

### 2.1 Mineral Synthesis and Characterization

The two types of synthetic periclase (MgO) nanocubes used for this study were made by calcinating synthetic brucite [Mg(OH)_2_] at 500 °C (Pe5) and at 1000 °C (Pe10) under atmospheric air for 2 h (Figures 1b,c and S1–S3, Table S1). These annealing temperatures were chosen because periclase nanocubes produced below and above threshold of 650 °C have contrasting properties.^14,15^ The resulting solids were then ground using a mortar and pestle, and powders were transferred to a N_2_(g)-filled glovebox (Omni-Lab System VAC; ∼18 ppm H_2_O, ∼8 ppm O_2_) for long-term storage to minimize exposure to the atmosphere. Synthetic Mg(OH)_2_ nanoparticles were produced by neutralizing a 0.2 M MgCl_2_ by 0.5 M NaOH under N_2_(g).

AMC and nesquehonite were also synthesized to compare with carbonation reaction products. AMC was synthesized by rapidly mixing equal volumes of 0.2 M MgCl_2_ and 0.2 M Na_2_CO_3_ solutions while stirring at 25 °C. The precipitate was then immediately centrifuged at 2900*g* for 10 min to separate supernatant and to prevent aging into nesquehonite. Nesquehonite was, in turn, synthesized by dropwise addition of equal volumes of 0.2 M Na_2_CO_3_ to 0.2 M MgCl_2_ solution while stirring at 25 °C. The resulting suspension was then aged for 48 h during which time it crystallized to nesquehonite. The wet centrifuged pastes were then dried at 25 °C under N_2_(g) before being ground to powder.

All synthetic materials were analyzed for (i) phase purity by X-ray diffraction (XRD; Figure S1), (ii) hydroxyl and carbonate composition by Fourier transform infrared (FTIR) spectroscopy, (iii) specific surface area and microporosity by N_2_(g) adsorption/desorption isotherms (Figure S2), (iv) particle size and geometry by scanning and by transmission electron microscopy (SEM, TEM; Figure S3), and surface composition by X-ray photoelectron spectroscopy (XPS; Table S1). XRD measurements were carried out using a PANalytical X’Pert^3^ instrument (Cu Kα radiation at 45 kV and 40 mV) operating under reflection mode. FTIR spectra were acquired using an attenuated total reflectance (ATR) cell (Golden Gate, Serial Number N29328 by Specac) with a FTIR spectrometer (Bruker Vertex 70/V) equipped with a deuterated l-alanine-doped triglycine sulfate (DLaTGS) detector. Brunauer–Emmet–Teller (BET) specific surface area, Barrett–Joyner–Halenda pore size, and volume were obtained from 90-point N_2_(g) adsorption/desorption isotherms. These isotherms were collected on samples previously degassed at 110 °C under a flow of N_2_(g) for 24 h using a Micromeritics TriStar 3000 instrument. SEM images were taken on a Carl Zeiss Merlin microscope. TEM images were taken with FEI Talos L120 microscope (120 kV). High-resolution transmission electron microscopy (HRTEM) images were taken under cryogenic conditions (−90 °C). These images were acquired with a FEI Titan Krios instrument equipped with a field emission gun operated at 300 kV and a K2 detector. Finally, XPS measurements were acquired using a Kratos Axis Ultra electron spectrometer equipped with an Al Kα X-ray source, 150 W, and a delay line detector. Survey spectra were collected from 0 to 1100 eV at a pass energy of 160 eV, while core level spectra of C 1s, O 1s, and Mg 2p were taken at 20 eV.

### 2.2 CO_2_ Mineralization by XRD

Phase changes caused by CO_2_ mineralization of MgO were tracked in situ by powder XRD. These measurements were performed in transmission mode using an Anton Paar MHC-trans humidity chamber. The sample stage was first aligned along the vertical wall using corundum powder as a standard. This procedure was conducted prior to all experiments to ensure that diffraction peaks did not shift from a misplaced sample stage. Periclase samples were thereafter placed on small cups assembled with a thin Kapton film at the bottom for incoming X-rays, after which they were dried with N_2_(g) (0% RH) at 30 °C for 60 min. The samples were then exposed to a flow of 250 mL/min N_2_(g) containing ∼2.0 kPa CO_2_ and 3.77 kPa H_2_O (90% RH) at 30 °C for a 20 h period. This humidified gas mixture was created by a humidity generator (ProUmid MHG32) using a mixture of 2% mol CO_2_ in 98% mol N_2_(g) (AirLiquide) as the carrying gas. Diffractograms were continuously collected in the 10–55° 2θ range during the reactions. All measurements were performed using a PANalytical X’Pert^3^ powder diffractometer, and XRD profiles were analyzed using the GUI software of Profex v4.1.

### 2.3 CO_2_ Mineralization by Vibrational Spectroscopy

Carbonate species and water films formed during the reaction were monitored in situ by FTIR spectroscopy. Reactions were conducted in the 20–90% RH range at 25 °C and at 90% RH in the 30–80 °C range. Pe5 and Pe10 particles were applied on the diamond window of an ATR (diamond, single-bound; Golden Gate, Serial Number N29328 by Specac) cell in the form of pastes centrifuged from ethanol suspensions. Ethanol was chosen to prevent hydrolysis or carbonation reactions that could otherwise be triggered by water. The pastes were covered by a closed flow-through PEEK-lined stainless-steel lid and were dried under N_2_(g) for at least 60 min at 25 °C. Continual monitoring the FTIR spectra confirmed the complete removal of ethanol (C–O stretching modes 1055 and 1102 cm^–1^).

The resulting solid-state MgO films were exposed to a flow of 507.6 mL/min 101 kPa N_2_(g) mixed with a predetermined partial pressure of water vapor in the 0–2.75 kPa range (0–90% RH at 25 °C) H_2_O and ∼2.0 kPa (20,000 ppm) CO_2_(g). We chose this CO_2_ pressure to facilitate carbonation reactions under an experimentally feasible time frame from minutes to hours. This gas mixture was continuously monitored for composition using a non-dispersible infrared device (LI-7000, Licor Inc). It was prepared by mixing a 7.6 mL/min flow of dry CO_2_(g) with a 500 mL/min flow of water vapor using mass flow controllers (MKS, 179A) to achieve a total flow of 507.6 mL/min. This moist gas was, in turn, prepared by mixing predetermined proportions of humid and dry N_2_(g) using a humidity generator module (proUmid MHG32). In an additional set of experiments, deuterium exchange reactions were triggered by exposing a sample to D_2_O(g) directly after it was reacted to 2.0 kPa CO_2_(g) under 90% RH (H_2_O). Deuterated water vapor was generated by passing 101 kPa of CO_2_-free N_2_(g) through a 316 stainless-steel flow-through cylinder containing D_2_O liquid (99.9%). This saturated D_2_O vapor was passed through a mass controller (MKS, 179) to generate a constant flow of 200 standard cubic square centimeters of ∼1.0 kPa D_2_O(g) (13% RH at 25 °C).

All time-resolved FTIR spectra were collected in the 600–4000 cm^–1^ range using a spectrometer (Bruker Vertex 70/V) equipped with a deuterated l-alanine-doped triglycine sulfate (DLaTGS) detector. Each spectrum was collected over 89 s period and was the average of 100 scans collected at a 4 cm^–1^ resolution and at a forward/reverse scanning rate of 10 kHz.

Finally, to resolve the time-dependent speciation of carbonate and relative water loadings, we deconvoluted the 1200–1900 cm^–1^ region using Gaussian components
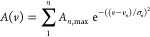
1Here, the wavenumber-dependent absorbances [*A*(*v*)] were resolved in terms of linear combinations of *n* individual Gaussian components, each with their maximal absorbance (*A*_*n*,max_), centered at wavenumber *v*_*n*_, distribution width σ_*n*_. All absorbances were normalized to that of the unreacted periclase at 667 cm^–1^ to account for variations in sample mass. All calculations were performed with MATLAB (version R2021b, The Mathworks, Inc.).

### 2.4 Ex Situ Characterization

Reaction products were also characterized ex situ by TEM to image particles, XPS to evaluate the surface composition of the reaction products, and thermal decomposition to compare the thermal stabilities of the reaction products and reference solids. Samples were prepared by reacting aliquots of ∼500 mg Pe5 and Pe10 powders in closed-loop glass vials. These vials were exposed to a stream of 507.6 mL/min N_2_(g) containing 0–2.75 kPa H_2_O and ∼2.0 kPa CO_2_(g) for 0, 5, 13, and 20 h. The resulting samples were then imaged by TEM using a FEI Talos L120 microscope (120 kV). Surface elemental compositions of samples reacted for 20 h were also measured by XPS using a Kratos Axis Ultra electron spectrometer equipped with a delay line detector. This instrument was equipped with a monochromatic Al Kα radiation source operated at 150 W, a hybrid lens system with a magnetic lens, as well as a charge neutralizer. Finally, water and carbonate thermal decomposition temperatures and loadings were quantified by thermogravimetric analysis (TGA, Mettler Toledo). These findings were obtained by heating samples from 30 to 700 °C at a rate of 10 °C/min under a flow of 20 mL/min N_2_(g).

## 3 Results and Discussion

### 3.1 AMC Nanocoating Formation on MgO Nanocubes

**Figure 2:**
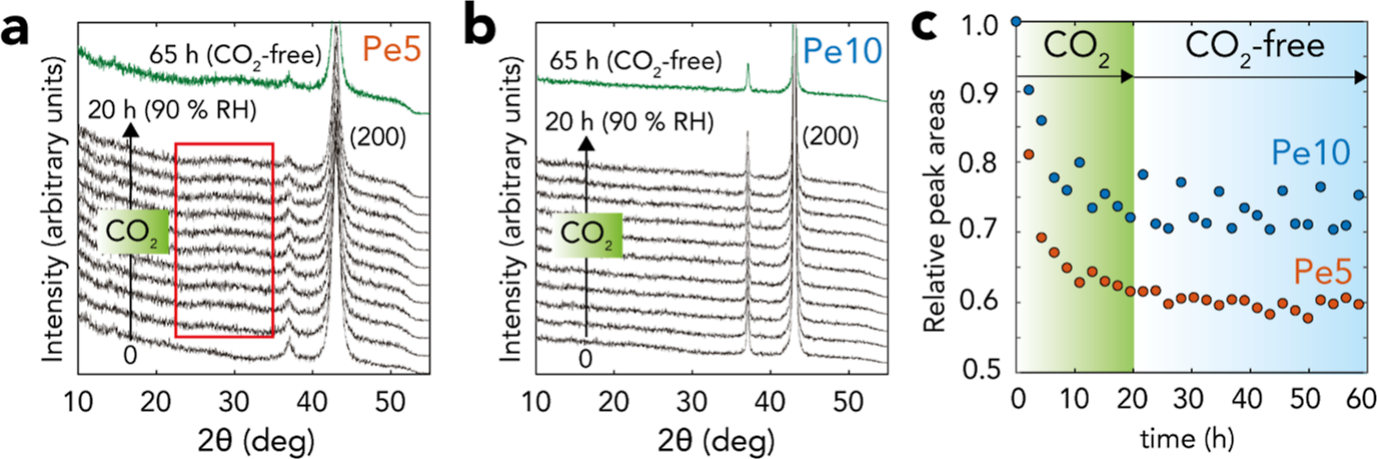
Water film-driven carbonation of MgO. XRD of (a) Pe5 and (b) Pe10 revealed systematic losses of the periclase content during exposure to 2.0 kPa CO_2_ with 90% RH in N_2_(g) at 25 °C. This was seen through the (c) relative areas of the (200) reflection (43° 2θ), normalized for area prior to the onset of the reactions. The red box in (a) highlights faint evidence for a new reflection, possibly from a carbonated product from reactions with Pe5. (c) Exposing the reacted samples for another 40 h in CO_2_-free 90% RH in N_2_(g) did not significantly consume periclase, suggesting that carbonated products formed nanocoatings blocking access to reactive species. Note that peak intensities cannot be used to precisely quantify proportions of converted periclase because diffraction peak intensities were not linearly proportional to the phase concentration.

**Figure 3:**
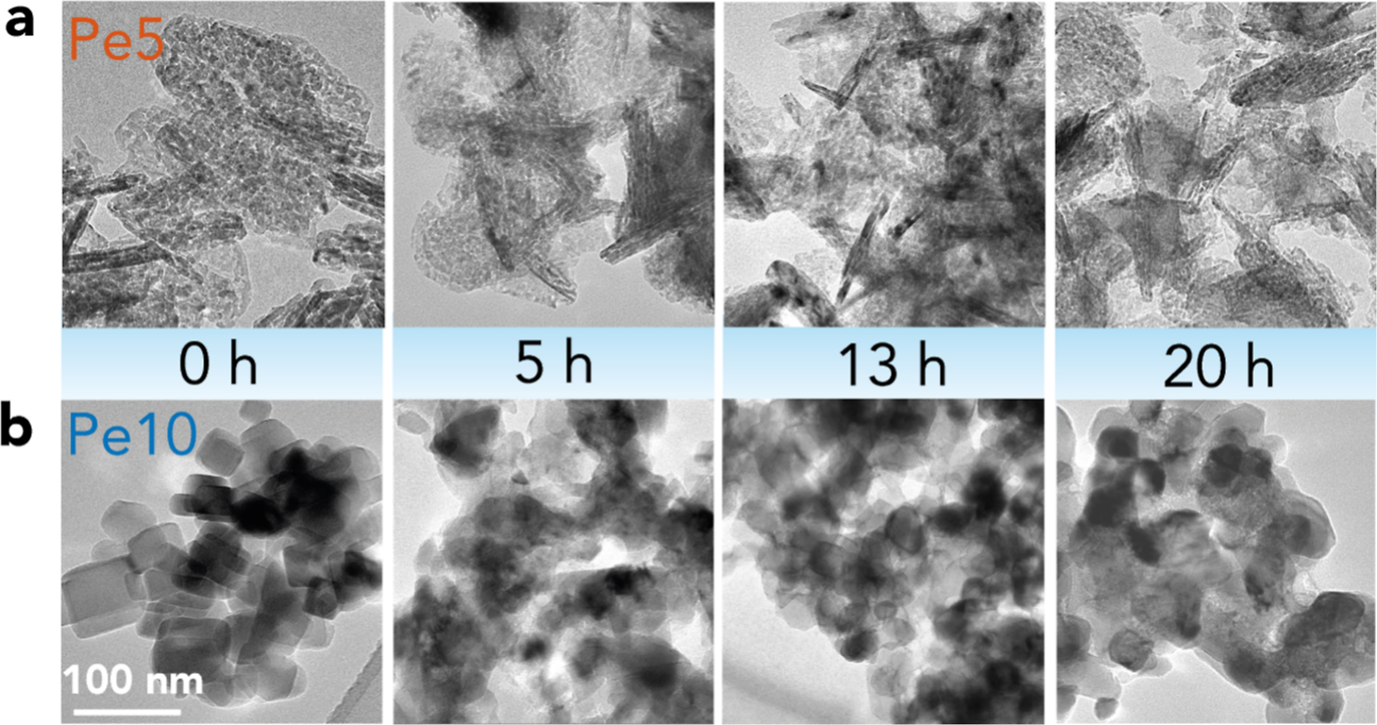
TEM images showing time-resolved sequence of reacted (a) Pe5 and (b) Pe10 under a stream of 90% RH H_2_O and 2.0 kPa CO_2_ for up to 20 h.

**Figure 4:**
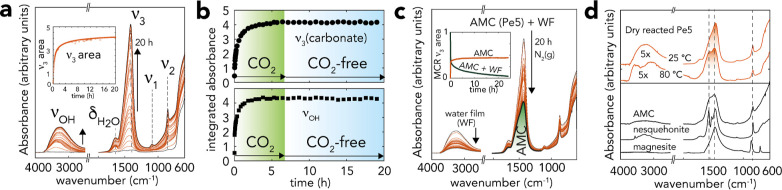
(a) Vibrational spectroscopy revealed AMC growth on Pe5 exposed to 2.0 kPa CO_2_ with 90% RH for up to 20 h. The inset shows the integrated area of the ν_3_ band of carbonate. See Figures S5–S7 for full data set, including for Pe10. (b) Band area of ν_3_ (carbonate) and of the entire ν_OH_ in Pe5 exposed to 2.0 kPa CO_2_ with 90% RH for up 7 h, and subsequently to CO_2_-free 90% RH for another 13 h (Figure S8 for spectra). (c) 20 h drying period under N_2_(g) after the 20 h reaction in (a), revealing the loss of about half of the carbonate, and a substantial portion of the water film. The inset shows the concentration profiles of the wettest (AMC + WF) and the driest (AMC) sample over reaction time. These profiles were extracted by multivariate curve resolution (MCR) analysis.^47^ See Figure S9 for full data set. (d) Vibrational spectra of the 20-h-reacted Pe5 (from (a)) dried under N_2_(g) at 25 °C and further to 80 °C (Figure S9) compared to those from synthetic materials: AMC, nesquehonite (MgCO_3_·3H_2_O), and magnesite (MgCO_3_). The spectrum of magnesite was generated using data from the RRUFF project (ID: R050676).^48^

**Figure 5:**
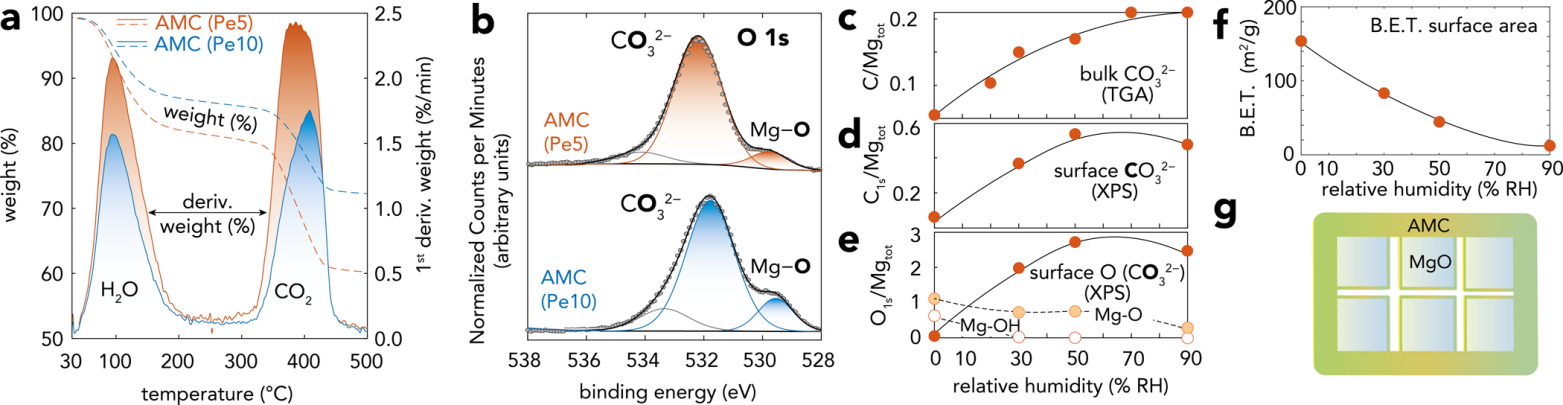
(a) TGA-derived chemical composition of reacted Pe5 and Pe10 under 2.0 kPa CO_2_ with 90% RH for 20 h. Mass of water (17.3% w/w for Pe5, 12.5% w/w for Pe10) and CO_2_ (∼400 °C, 22.7% for Pe5 and 13.3% for Pe10) revealed the MgCO_3_·2.3–2.5H_2_O composition of AMC. (b) XPS O 1s region of reacted AMC surfaces of same samples analyzed by (a) TGA, revealing dominance of carbonate O speciation (cf. Figure S11 for C 1s region). Underlying, unreacted MgO is seen in through Mg–O species at 539.6 eV (5.47 at. % on Pe5; 8.29 at. % on Pe10). (c–e) Humidity-resolved (c) TGA-derived bulk and (d,e) XPS-derived surface composition of Pe5 reacted under 2 kPa CO_2_ in the 0–90% RH range. Results revealed an enrichment of carbonate species at sample surfaces with respect to the bulk. (f) BET specific surface area (m^2^/g) of Pe5 reacted in 2 kPa CO_2_ with 30–90% RH for 20 h. (g) Idealized schematic representation of AMC nanocoatings responsible for important drop in BET specific surface area. Lines in (c–f) are visual guides to the trend outlined by the data points.

To begin exploring CO_2_ mineralization reactions, we exposed Pe5 and Pe10 nanocubes to a stream of 2.0 kPa CO_2_ with 90% RH at 25 °C, which produced ∼3–4 ML thick water films (Figure S4). The carbonated reaction products were analyzed by XRD (Figure 2), TEM (Figure 3), vibrational spectroscopy (Figure 4), TGA (Figure 5), and XPS (Figure 5).

In situ XRD measurements revealed a systematic consumption of periclase during the reactions (Figure 2a,b). Still, no crystalline materials clearly formed, saving faint evidence for a new broad reflection at ∼30° 2θ from an unresolved phase (Figure 2a, red box). From the main (200) reflection of MgO, we estimate that reactions after 20 h consumed only ∼40% of Pe5 and ∼30% of Pe10 (Figure 2c). We note that this is only an estimate as XRD peak intensities are not necessarily linearly proportional to sample mass. These results notably contrast with our recent work^12^ where reactions in a stream of CO_2_(g)-free 90% RH completely converted Pe5 and ∼80% of Pe10 to crystalline brucite within 20 h. We interpret the slower periclase consumption after ∼10 h by the formation of an AMC nanocoatings, forming a core–shell structure of the type MgO@AMC. These noncrystalline coatings blocked access of water and carbonate ions to the reactive MgO core. This interpretation was confirmed further in a second stage of the experiment where exposure to a CO_2_(g)-free moist N_2_ (Figure 2a–c) did not produce brucite, given by the absence of brucite reflections and the stable intensity of periclase reflections after this additional exposure.

TEM imaging (Figure 3) revealed that the reactions formed new coexisting solids with the Pe5 and Pe10. These new solids could, however, only be observed for a few minutes as they were readily decomposed under the high energy electron beam of the microscope.^46^ Additionally, because of the insufficiently large contrast in density between MgCO_3_ and MgO, TEM images could not clearly be used to resolve the carbonate-oxide core–shell structure inferred by XRD. Still, these images support XRD results showing only a partial consumption MgO nanocubes and, for the case of the larger Pe10 nanocubes, these revealed a clear transformation loss in the particle morphology (Figure 3b).

A direct confirmation that the nanocoatings were carbonated products of MgO was provided by vibrational spectroscopy (Figure 4a–c for Pe5, Figures S5–S7 for full data set for Pe5 and Pe10). Reactions in a stream of 2.0 kPa CO_2_(g) with 90% RH generated (i) C–O stretching (ν_1_, ν_2_, and ν_3_) bands from carbonate, as well as (ii) water bending (δ_H_2_O_) and stretching (ν_OH_) bands (Figure 4a,b). Both sets of bands evolved in tandem, suggesting that water was concomitantly captured during carbonation reactions. Consistent with the XRD-resolved consumption of periclase (Figure 2c), carbonation reactions induced by CO_2_(g) in 90% RH were fast in the initial stages of the reaction but slowed down after 4–5 h (*cf.* inset of Figure 4a). In another experiment, AMC grown for 7 h did not produce brucite (*i.e*., no ∼3700 cm^–1^ band), or related Mg(OH)_2_ precursors were recently resolved,^20^ when it was subsequently exposed to a CO_2_(g)-free moist air (Figure 4c). This confirmed that the AMC nanocoatings blocked diffusion of reactive species to the MgO core, as inferred by XRD (Figure 2).

To confirm the identity of the carbonate solid formed on MgO, we dried the reaction product under a flow of N_2_(g) (Figures 4c and S9). This procedure removed an important fraction of film water molecules and dissolved, unreacted, carbonate. It consequently revealed the spectral signature of AMC.^22,24,49^ The absence of the fine band structure of nesquehonite or of the single of magnesite supports the interpretation that carbonate ions and hydration water molecules were not in crystallographic positions. Based on recent work^23,24^ that linked the local structure of AMC to those of nesquehonite and hydromagnesite, it is likely that the AMC carbonate ions were also asymmetrically coordinated to three MgO_6_ units and surrounded by crystalline hydration water molecules. In the next section, we build upon these findings to explore how variations in water film coverage affect AMC bulk and surface composition, as well as growth rates.

### 3.2 Humidity-Resolved AMC Growth

To begin resolving the response of AMC growth to water film coverages, we analyzed the bulk (Figure 5a) and surface (Figure 5b, Tables S1 and S2) composition of MgO samples reacted to moist CO_2_(g) between 20 and 90% RH. Using microgravimetry to measure water loadings^43^ (Figure S4), we find that the onset of the reactions took place in sub-ML films below 50% RH and up to 3–4 MLs at 90% RH. TGA (Figure 5a) of samples reacted for 20 h revealed that C/Mg_tot_ ratios directly scaled with humidity, with values plateauing at C/Mg_tot_ = ∼0.2 at ≥ 70% RH (Figure 5c). Still, nanocoatings formed between 30 and 90% RH had the expected^21,23,24^ MgCO_3_·2.3–2.5H_2_O composition of AMC, regardless of humidity. This result thus aligns with the simultaneous growth of δ_H_2_O_ and ν_OH_ bands by water vapor capture both into the AMC bulk and at AMC surfaces. Additionally, a deuteration experiment (Figure S10) showed that all AMC water molecules were readily accessible for exchange. XPS (Figures 5b,d,e and S11) showed, in turn, that the topmost portions of the sample surfaces were considerably more enriched in carbonate (C/Mg_tot_ = 0.6) than the overall TGA-derived bulk. This supplied additional evidence that the reactions generated a core–shell structure of the type MgO@AMC. XPS also detected Mg-bound O from the unreacted MgO core in all reacted samples (Figure 5b,e, and Tables S1 and S2), a result signaling that nanocoatings were thinner than the analysis depth (∼5–10 nm) of the technique. All Mg–OH species were, on the other hand, completely reacted as these were from surface functional groups.

Using N_2_(g) adsorption/desorption, we also found that the BET specific surface area^50^ of the reacted Pe5 was systematically lowered with humidity (Figure 5f). Because this drop in surface area also aligned with AMC nanocoating coverage (Figure 5c–e), these results suggest that AMC nanocoatings must have effectively encapsulated several (partially reacted) particles (*e.g.*, Figure 5g). These nanocoatings must have, as such, been sufficiently impermeable to even block N_2_(g) diffusion into the underlying MgO nanocubes.

**Figure 6:**
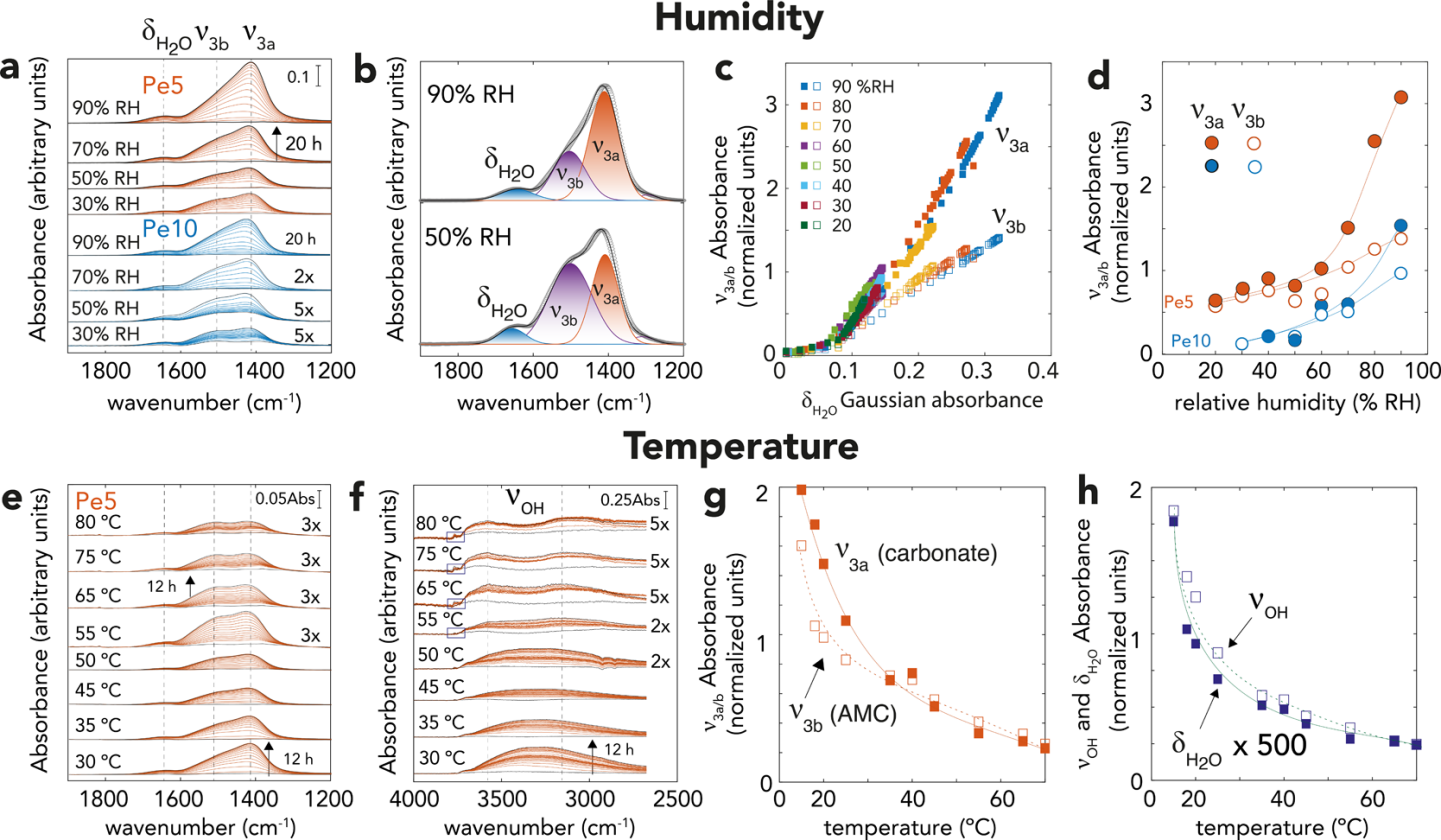
Vibrational spectroscopy revealing MgO carbonation over a range of (a–d) humidity and (e–h) temperature. (a) FTIR spectra of Pe5 and Pe10 reacted in 2.0 kPa CO_2_ and 30–90% RH at 25 °C. (b) Examples of Gaussian deconvolution of selected Pe5 spectra of the 1200–1900 cm^–1^ region in two distinct C–O stretching (ν_3a_, ν_3b_) bands and in one water bending (δ_H_2_O_) band. Spectra were not scaled. (c,d) Relationship between *A*_*n*,max_ (eq 1) absorbances of carbonate (ν_3a_, closed symbols; ν_3b_, open symbols) and (c) water (δ_H_2_O_) Gaussian components at all stages of the reaction for Pe5, and (d) relative humidity (% RH) after 20 h of reaction for Pe5 (orange) and Pe10 (blue). (e,f) FTIR spectra of Pe5 reacted in 2.0 kPa CO_2_ and 2.4 kPa H_2_O (80% RH at 25 °C). Small black boxes in (f) outline MgO surface OH groups indicated sub-ML films coverage (*cf.*Figure S7 for close-up view). (g) Relationship between *A*_*n*,max_ (eq 1) absorbances of ν_3a_ (closed symbols) and ν_3b_ (open symbols) and temperature in samples reacted for 12 h. (h) Relationship between *A*_*n*,max_ (eq 1) absorbances of δ_H_2_O_ and integrated band area of the O–H stretching region (ν_OH_) with temperature in samples reacted for 12 h. Note all absorbances were normalized to that of the unreacted periclase at 667 cm^–1^ to account for variations in sample mass.

Building upon these results, we tracked the humidity-resolved growth of AMC using its main C–O stretching (ν_3_) band (Figure 6a). While this C–O stretching region of carbonate was in the form of a singlet in films of more than 2 MLs (70–90% RH), it developed into a doublet when AMC grew in films of less than 2 MLs (20–60% RH). To better capture these changes, we deconvoluted this spectral region (Figure 6b) using two Gaussian-shaped (eq 1) components (ν_3a_ = 1410 ± 2 cm^–1^ and ν_3b_ = 1503 ± 1 cm^–1^), and we used one component to resolve the partially overlapping bending band of water (δ_H_2_O_ = 1649 ± 7 cm^–1^). This procedure revealed that ν_3a_ and ν_3b_ component absorbances (*A*_*n*,max_, eq 1) directly correlated with water film coverages (Figure 6c,d) during all stages of the reaction. It also showed that ν_3a_ band preferentially grew in films thicker than ∼2 ML (i.e. >60% RH). This band was however also preferentially removed during dehydration experiments (Figures 3b and S9), leaving the signature doublet of AMC in the absence of free water.

We explain the preferential growth of the ν_3a_ component by the formation of solvated carbonate species co-existing with AMC solid in the thicker water films. This can be appreciated by the strong similarity in wavenumber and shape of the ν_3a_ component with that of the trigonal planar (*D*_3*h*_ symmetry) CO_3_^2–^ ion (Figure S12). Despite the absence of any clear spectroscopic signatures of bicarbonate, we are still open for the possible existence of bicarbonate species at this water coverage because thermodynamics (Supporting Information, Figure S13) predict a circumneutral pH (pH ∼ 8.2). Predictions of the open MgCO_3_(s)–CO_2_(g)–H_2_O(l) system (Figure S13), which we chose as a proxy to simulate water film chemistry, suggest MgHCO_3_^+^, HCO_3_^–^, MgCO_3_^0^, and CO_3_^2–^ ions as the dominant aqueous carbonate species. While the prime source of these soluble species was from CO_2_(g), additional hydration experiments in the absence of CO_2_(g) (Figure S14) showed that AMC dissolution was a secondary source for carbonate. Predictions of closed systems (Figures S13 and S15) however revealed that MgCO_3_^0^(aq) should be the dominant species, resulting from alkaline (pH ∼ 10.9) condition. Based on previous work^51^ showing strongly similar spectral signatures of the CO_3_^2–^ ion with MgCO_3_^0^(aq), we conclude that water films must have hosted a solvent-shared MgCO_3_^0^(aq) pair, implying that the carbonate ion retained its hydration environment. For these reasons, we foresee that the MgCO_3_^0^(aq) ion pair was the primary species formed during AMC dissolution under low pressure of CO_2_(g). The MgHCO_3_^+^, HCO_3_^–^, MgCO_3_^0–^ ions became, on the other hand, the dominant soluble species at higher CO_2_(g) pressures. The unclear evidence for bicarbonate ions can be explained by the (i) overlap of strong and dominant C–O signals from solid AMC, and (ii) the potentially smaller absorption coefficient of the ion compared with soluble CO_3_^2–^ (Figure S12), and (iii) the greater similarity of the spectral shape of ion pairs and free ions mixture to the spectrum of CO_3_^2–^.^51^ Therefore, given the response of the ν_3a_ component to the aqueous speciation of the water film, we conclude that the evolution of the ν_3b_ component is a more reliable marker for AMC growth than ν_3a_. This can, additionally, be appreciated by the comparable humidity-resolved response of the ν_3b_ component with TGA (Figure 5a,c) and XPS (Figure 5b,d,e) results.

We arrived to the same conclusion by tracking AMC growth at temperatures of up to 80 °C (Figure 6e,f). Here, experiments under a flow of 2.0 kPa CO_2_ and 2.4 kPa H_2_O (80% RH at 25 °C) showed that MgO surfaces were more dehydrated at high temperatures. This was appreciated by the loss of the water film O–H stretching band (∼3300 cm^–1^), and by the appearance of periclase surface OH groups at 3765 cm^–1^ above 50 °C (*cf*. boxed areas of Figure 6f are featured in Figure S7). The co-existence of these bands at high temperatures indicated that AMC growth reactions could have even proceeded in sub-ML water films. As in the humidity-resolved experiments at 25 °C (Figure 6a), these temperature-resolved experiments showed that the ν_3_ region evolved from a singlet in thick water films to a doublet in the thinner films formed at high temperature (Figure 6e). This can also be appreciated by the congruent temperature-resolved absorbances of the ν_3a_ and ν_3b_ (Figure 6g) with those of the water components (Figure 6h). From the absorbances (*A*_*n*,max_, eq 1) of the ν_3b_ band after 20 h, we estimate that ∼6–7 times less AMC formed at 80 °C than that at 25 °C. We can explain this important difference in the reaction yield by the combined effects of the lower (i) CO_2_ solubility and (ii) water loadings at high temperatures.

**Figure 7:**
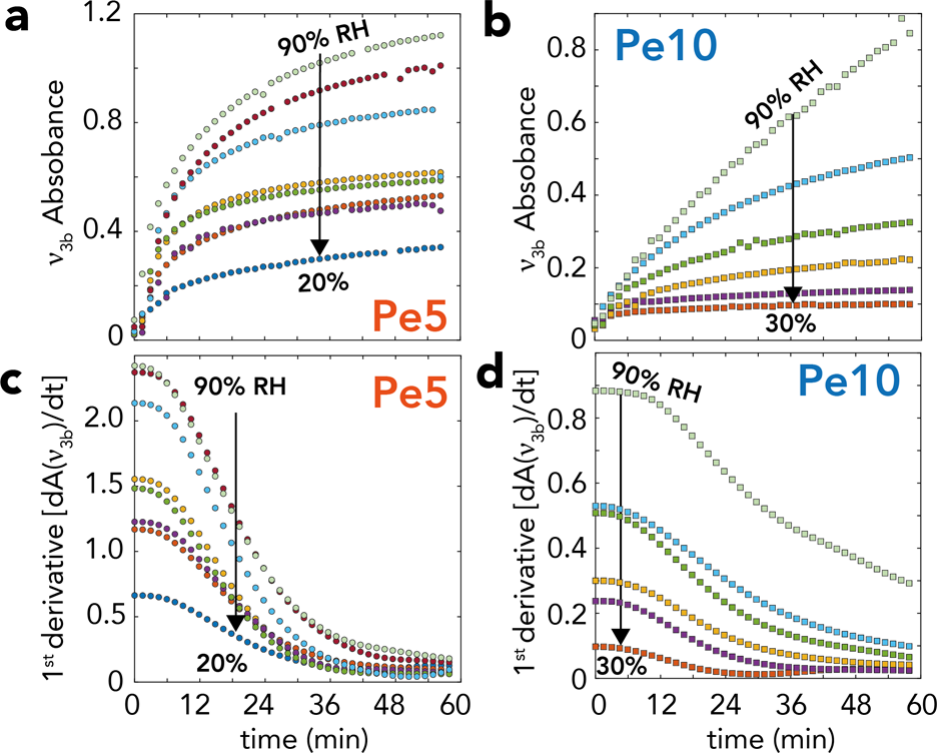
Time-resolved growth of AMC in (a,c) Pe5 and (b,d) Pe10 over ranges of humidity in the first 60 min of reaction (Figures S16 and S17 for all data up to 20 h of reaction). (a,b) Absorbances of the ν_3b_ component and (c,d) first derivative of these data, here used as a proxy for instantaneous rates. Rates were slower in Pe10 due to the lower specific surface area.

Next, to resolve the humidity- and temperature-dependent AMC growth kinetics, we analyzed the component band areas (*A*_*n*,max_, eq 1) of AMC (ν_3b_) and dissolved (bi)carbonate (ν_3a_) over reaction time (Figures 7 and S16 and S17). This analysis revealed that both AMC and dissolved (bi)carbonate loadings co-evolved over the course of the reactions (Figures S16 and S17), and that yields directly scaled with humidity and water loadings. It also provided insights into the kinetics of the reactions. To limit assumptions regarding complex reaction rate orders for metal oxide carbonation reactions,^52^ we used the first derivative values of absorbances (*A*_*n*,max_, eq 1) of AMC, which are proxies for rate (Figure 7c,d). This approach was motivated further by inconclusive chemical kinetic modeling attempts. Our analysis showed that growth rates scaled with water loadings (high humidity and low temperature) and were fastest within the first ∼10 min of reaction, after which they slowed down over the course of 30–45 min. AMC growth was subsequently replaced by a slower long-term regime over at least the course of 20 h. Rates were, additionally slower in Pe10 nanocubes (Figure 7d), and this can be understood by the smaller nanocube surface area. Following our previous work on MgO hydroxylation,^12^ these results were taken as indication that long-term carbonation reactions could also be explained, at least qualitatively, in terms of the shrinking core model (Figure 1a).^53^ Accordingly, the initial high growth rates of the ν_3b_ band can be explained by a short-lived solvent-driven process, in which (*e.g.*, nucleation-limited^54^) complexation reactions give rise to the incipient AMC nucleation clusters leading to growth. The slower, long-term, growth rate signals, on the other hand, a subsequent diffusion-limited regime, in which AMC nanocoatings hampered access of reactive species to the MgO core. Again, attempts at hydroxylating MgO in core–shell MgO@AMC solids (Figures 2c and 4b) showed AMC nanocoatings to be highly efficient diffusion barriers for efficient CO_2_ mineralization.

**Figure 8:**
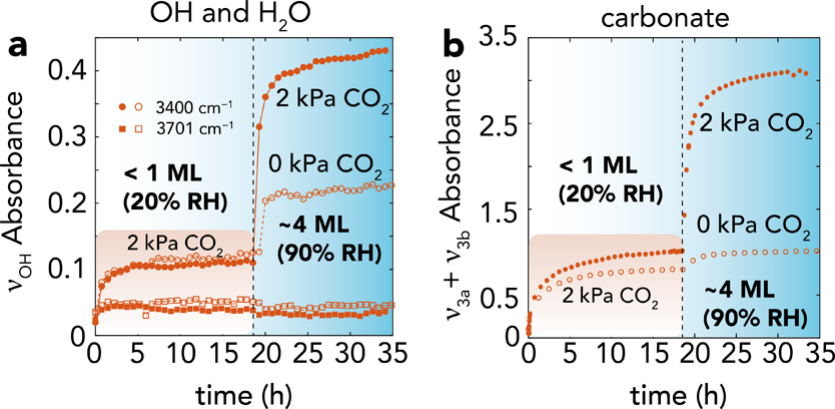
AMC partial dissolution-reprecipitation by exposure to thicker water films. Time-resolved growth of (a) O–H stretches, (b) and carbonate (ν_3a_ + ν_3b_) regions of Pe5, which was first reacted under a stream of 20% RH and 2.0 kPa CO_2_ for 19 h. The resulting MgO@AMC was then reacted under a stream of 90% RH with 0 kPa CO_2_ or 2.0 kPa CO_2_ over the next 16 h (*cf*. Figure S18 for spectra). (a) O–H stretching bands did not provide evidence for brucite (3701 cm^–1^) growth, while the water band (3400 cm^–1^) quadrupled under CO_2_-bearing gases, indicating AMC growth. (b) C–O stretching bands revealed AMC growth from pre-grown AMC under CO_2_-bearing gases, in contrast to CO_2_-free gases.

Finally, in an effort to explore paths that bypass the inhibitive effects of AMC nanocoatings, we considered a strategy to re-expose portions of the unreacted MgO core to the water film (Figures 8 and S18). To illustrate this possibility, we first exposed Pe5 to a stream of 20% RH and 2.0 kPa CO_2_(g) for 19 h. After forming the AMC nanocoating in these sub-ML water films, the resulting products were exposed to CO_2_-free 90% RH. Based on changes in band absorbances, we estimate that water coverages doubled but that, from the absence of brucite (3701 cm^–1^), AMC nanocoatings effectively blocked access of water to the MgO core. However, exposing the same products to CO_2_-bearing 90% RH quadrupled water (Figure 8a) and tripled carbonate (Figure 8b) absorbance bands, thus indicating that carbonation reactions were again possible.

These results consequently revealed the possibility of bypassing the diffusion-limited regime established during AMC growth. We ascribe this to the partial dissolution of AMC (p*K*_s_ = 4.54–5.48)^55−57^ by slightly acidic CO_2_-rich water films, namely through a reaction of the type
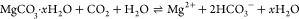
2

As such, by re-exposing the MgO core to CO_2_-rich water films, this process drove additional AMC growth. In the specific case considered here, exposing 90% RH to AMC grown at 20% RH prolonged the reaction for another 5 h, at which point AMC nanocoatings again slowed down the reaction. From the inherent acidity of CO_2_-bearing solution, increasing water coverages over AMC nanocoatings should inexorably promote the AMC growth. Knowledge of this possibility should be of especial interest for taking advantage of variations in air moisture in applications of CO_2_ mineralization.

## 4 Conclusions

This study monitored CO_2_ mineralization of MgO nanocubes hosting CO_2_-rich water films. We showed that AMC grows in sub-ML to multilayered water films with rates and yields scaling with water coverage.

Reactions produced, additionally, ∼6–7 times less AMC formed at 80 °C than that at 25 °C, a result of the compounded effects of the lower (i) CO_2_ solubility and (ii) water loadings at high temperatures.

AMC growth produced to nanocoatings were so effective at blocking reactivity that they prevented the full conversion of ∼8 nm wide Pe5 nanocubes. These films even inhibited the growth of hydroxide-bearing phases when exposed to water-rich, CO_2_-deficient air. These nanocoatings can, however, be partially dissolved by exposure to thicker CO_2_-bearing water films, a process that prolongs the reaction for several more hours until nanocoatings blocked the reactions again. These findings consequently provide evidence that CO_2_ mineralization by MgO-bearing products can face a potential limitation in the carbonation efficiency. These findings can have also direct implications in the study of carbonation reactions in related nanominerals (*e.g.*, CaO and FeO), as well as in the study of mineral growth in nanometrically thick water films.

## Supporting Information Available

X-ray power diffraction of periclase, N_2_(g) adsorption/desorption isotherms and microporosity analyses, electron microscopy imaging of periclase, microgravimetrically derived water loadings, full FTIR data set, additional FTIR experiments for deuterium exchange and dehydration, and XPS data (PDF)
