# Supplementary material for: CO2 Mineralization by MgO Nanocubes in Nanometric Water Films
Source: ACS Appl Mater Interfaces. 2023 Sep 14;15(38):45055–63. doi: 10.1021/acsami.3c10590 (PMC10540135; doi:10.1021/acsami.3c10590)
Supplement: Supplementary file 1 — am3c10590_si_001.pdf [file am3c10590_si_001.pdf]

Supporting Information

CO_2 mineralization by MgO nanocubes in nanometric water films

*N. Tan Luong, Noémie Veyret, Jean-François Boily**

Department of Chemistry, Umeå University, SE 901 87 Umeå, Sweden
*corresponding author: jean-francois.boily@umu.se

TABLE OF CONTENTS

SUPPORTING RESULTS 2
FIGURES 4
- Figure S1. 4
- Figure S2. 4
- Figure S3. 5
- Figure S4. 5
- Figure S5. 6
- Figure S6. 6
- Figure S7. 7
- Figure S8. 7
- Figure S9. 8
- Figure S10. 8
- Figure S11. 9
- Figure S12. 9
- Figure S13. 10
- Figure S14. 10
- Figure S15. 11
- Figure S17. 12
- Figure S18. 12

TABLES 13
- TABLE S1 13
- TABLE S2 13
- TABLE S3 14
- TABLE S4 14

REFERENCES 15

SUPPORTING RESULTS

Synthetic MgO nanocubes

The periclase nanocubes chosen to track carbonation reactions were calcinated ($Mg(OH)_2 \rightarrow MgO + H_2O$) at 500 ºC (Pe5) and at 1000 ºC (Pe10) from the same synthetic brucite nanoparticles.[1-3] These two calcination temperatures produced periclase nanocubes of contrasting crystallinity, particle size and aggregation modes.[4-10]

Pe5 nanocubes were of lower crystallinity (Fig. S1), of smaller Scherrer crystallite size (8.2 ± 0.4 nm for Pe5; 31.7 ± 4.2 nm for Pe10), larger specific area (154 m^2/g for Pe5; 25.7 m^2/g for Pe10) and even larger microporosity (1.36 cm^3/g for Pe5; 0.12 cm^3/g for Pe10) than Pe10 nanocubes (Fig. S2). Pe5 nanocubes were, additionally aggregated as nanobars within hexagonal casings (77 ± 25 nm wide) of the incipient brucite nanoparticles from which they were produced (Fig. S3).[11] These nanobars were also separated by slit-shaped pores likely lying along the (111) plane, producing a maze-like environment.[12, 13] Finally, Pe10 nanocubes were monodispersed and had comparable TEM particle (36.7 ± 10.6 nm) and Scherrer crystallite sizes (Fig. S3).

Thermodynamic calculations of equilibrium pH

1. **$MgCO_3$ equilibrium in pure water (close system).**

Reactions:

$MgCO_3(s) \rightleftharpoons Mg^{2+} + CO_3^{2-}$ $\qquad \log K_{AMC} = -5$ $\qquad$ (1) [14-16]

Assuming that hydrolysis of Mg^{2+} is negligible. The pH of the system is controlled by carbonate speciation.

Protonation:

$CO_3^{2-} + H_2O \rightleftharpoons HCO_3^{-} + OH$ $\qquad K_{a2} = 10^{-10.33}$ $\qquad$ (2)[17]

$HCO_3^{-} + H_2O \rightleftharpoons H_2CO_3^{*} + OH$ $\qquad K_{a1} = 10^{-6.35}$ $\qquad$ (3)[17]

Note: $H_2CO_3^*$ represents $CO_2(aq) + H_2O$

Proton condition (electroneutrality):

$2[Mg^{2+}] + [H^+] = [OH^-] + [HCO_3^-] + 2[CO_3^{2-}]$ $\qquad$ (3)

solubility product: $[Mg^{2+}] = K_{sp}/[CO_3^{2-}]$

Ionization fractions: $[CO_3^{2-}] = C_T\alpha_2$

$[HCO_3^-] = C_T\alpha_1$

With $\alpha_2 = 1/(1 + [H^+]/K_2 + [H^+]^2/K_1K_2)$

$\alpha_1 = 1/([H^+]/K_1 + 1 + K_2/[H^+])$

Then: $[Mg^{2+}] = K_{sp}/C_T\alpha_2$

Assume that all Mg^{2+} that become dissolved must equal in concentration the sum of the dissolved carbonic species, that is: $Mg^{2+} = C_T$

Then, $\quad [Mg^{2+}] = C_T = (K_{sp}/\alpha_2)^{0.5}$

Replace in the proton condition:

$2(K_{sp}/\alpha_2)^{0.5} + [H^+] = [OH^-] + \alpha_1 (K_{sp}/\alpha_2)^{0.5} + 2\ \alpha_2 (K_{sp}/\alpha_2)^{0.5}$

Thus we get:

$(K_{sp}/\alpha_2)^{0.5} (2 - \alpha_1 - 2\alpha_2) + [H^+] - K_w/[H^+] = 0$ $\qquad$ (4)

Using MATLAB to solve the equation (4), we find an equilibrium pH of ~10.888.

2. $MgCO_3$ equilibrium in CO_2-rich water (opened system).

pCO_2 = 2.6 kPa, $\log CO_2$ = -1.7

Reactions:

$MgCO_3(s) \rightleftharpoons Mg^{2+} + CO_3^{2-}$ $\log K_{AMC} = -5$ (1)

Assuming that hydrolysis of Mg^{2+} is negligible. The pH of the system is controlled by carbonate speciation. Because CO2 is in equilibrium with the system, it is convenient to write dissociation of carbonic acid instead of protonation:

$CO_2(g) \rightleftharpoons CO_2(aq)$ $K_H = 3.3\text{x}10^{-7}$ (5)[17]

$CO_2(aq) + H_2O \rightleftharpoons HCO_3^- + H_3O^+$ $K_{a1} = 10^{-6.35}$ (3)

$HCO_3^- + H_2O \rightleftharpoons CO_3^{2-} + H_3O^+$ $K_{a2} = 10^{-10.33}$ (2)

It is easier by summing up these equations into this equilibrium:

$MgCO_3(s) + CO_2(g) + H_2O \rightleftharpoons Mg^{2+} + 2HCO_3^-$ (6)

The proton condition in this case is still similar as in close system:

$2[Mg^{2+}] + [H^+] = [OH^-] + [HCO_3^-] + 2[CO_3^{2-}]$ (3)

Note: $[H_3O^+]$ can be simplified as H^+.

However, the assumption can be made to simplify the equation by taking in account that pH is controlled not only speciation of soluble carbonates from the solid, but also from $CO_2(g)$. The proton condition can be reduced by assuming negligible $[H^+]$ and $[OH^-]$ from water dissociation. Because the system is in equilibrium with $CO_2(g)$, the reaction (3) and (5) govern pH of the system.

(3) can be simplified as:

$2[Mg^{2+}] = [HCO_3^-]$ (7)

Or $\log[Mg^{2+}] = \log[HCO_3^-] - 0.3$ (8)

Eq. (8) can be solved graphically by Fig. S13.The intersection of lines $\log[Mg^{2+}]$ and $\log[HCO_3^-] - 0.3$ gives pH ~8.1-8.3 at equilibrium condition.

To solve Eq. (8) exactly, we consider these expressions:

$K_{sp} = [Mg^{2+}][CO_3^{2-}]$ (9)

$K_H = [CO_2]/pCO_2$ (10)

$K_{a1} = [HCO_3^-][H^+]/[CO_2]$ (11)

$K_{a2} = [CO_3^{2-}][H^+]/[HCO_3^-]$ (12)

Combining (9)-(12) into (7):

$[H^+] = (K_H^2 K_{a1}^2 K_{a2} (p_{CO2})^2 / 2K_{SP})^{1/3} = ((3.3\text{x}10^{-7})^2(10^{-6.35})^2(10^{-10.33})(2600)^2 / 2(10^{-5}))^{1/3} = 10^{-8.15}$

At equilibrium pH = 8.15.

FIGURES

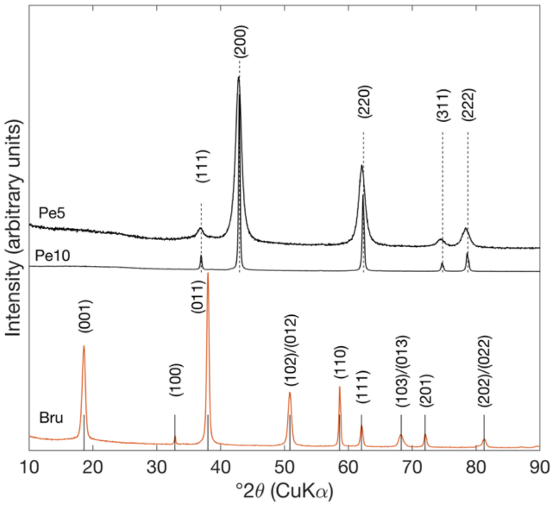

Figure S1. XRD profiles of periclase (Pe5 and Pe10) and brucite (Bru) taken in reflection mode. Reference lines of periclase (dash, AMCSD 0000501)[18, 19] and brucite (solid, AMCSD 0007912)[18, 20] are shown for comparison. Note that the relative intensities of these reference lines were neglected in the plot.

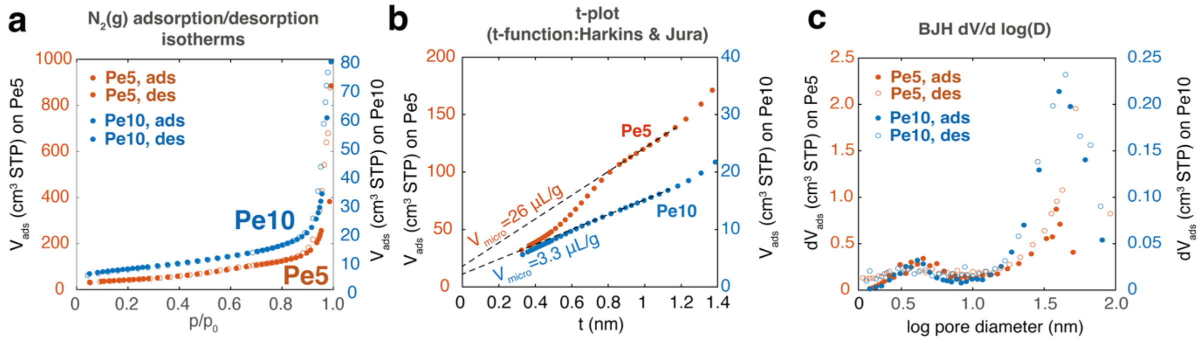

Figure S2. (a-c) N_2(g) adsorption/desorption isotherm results showing (a) raw data, (b) t-plots and (c) BJH analyses. These revealed specific surface area on par with particle sizes, and microporosity of 26 mL/g in Pe5 but of only 3.3 mL/g Pe10. We assign this microporosity to interparticle voids, given their comparable distributions of values in both Pe5 and Pe10 nanocube assemblages.

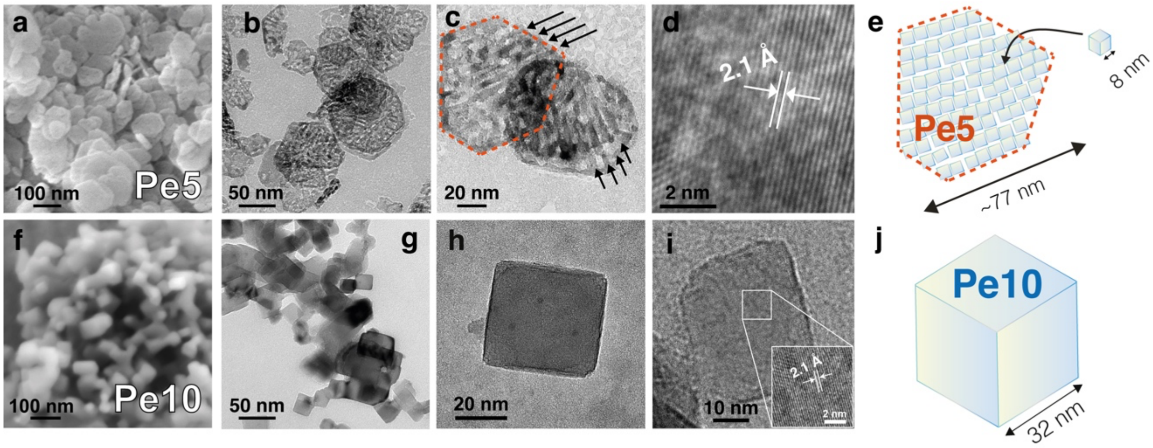

Figure S3. Periclase nanocube morphology and size. Electron microscopy images of (a–d) Pe5 and (f–i) Pe10, and (e, j) corresponding schematic representations. Scanning Electron Microscopy (a, f) and Transmission Electron Microscopy revealed (b-c) Pe5 nanocubes clustered as nanobars in hexagonal casings, which are relicts of the synthetic brucite from which they were produced. Arrows in (c) highlight preferential arrangement of the ~8-nm wide Pe5 nanocubes into nanobars in a fashion aligning with previous work [21, 22]. (g–h) Pe10 nanocubes were monodispersed. (d, i) High Resolution Transmission Electron Microscopy revealed diffraction fringes expected from the crystallographic structure of periclase.

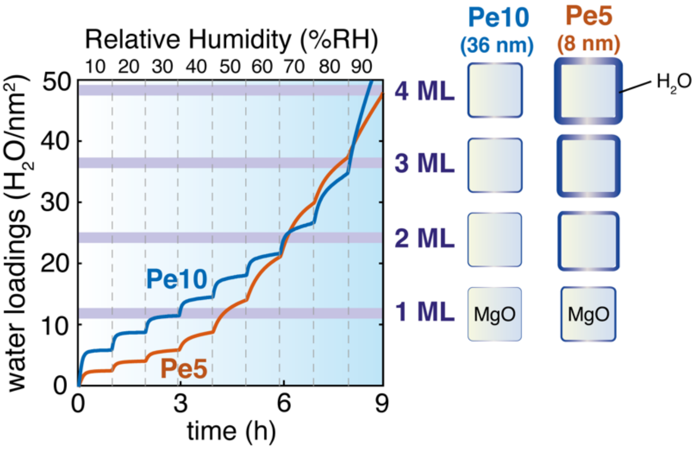

Figure S4. Microgravimetrically-measured water loadings by exposing a flow of water vapor from 0.9 to 92 % RH at 10 % RH intervals, each with a reaction time of 1 h. The right-hand side of is a size-scaled schematic representation of the total equivalent water films thickness in relation periclase nanocube size. One water monolayer (ML) corresponds to 12 H_2O/nm^2.

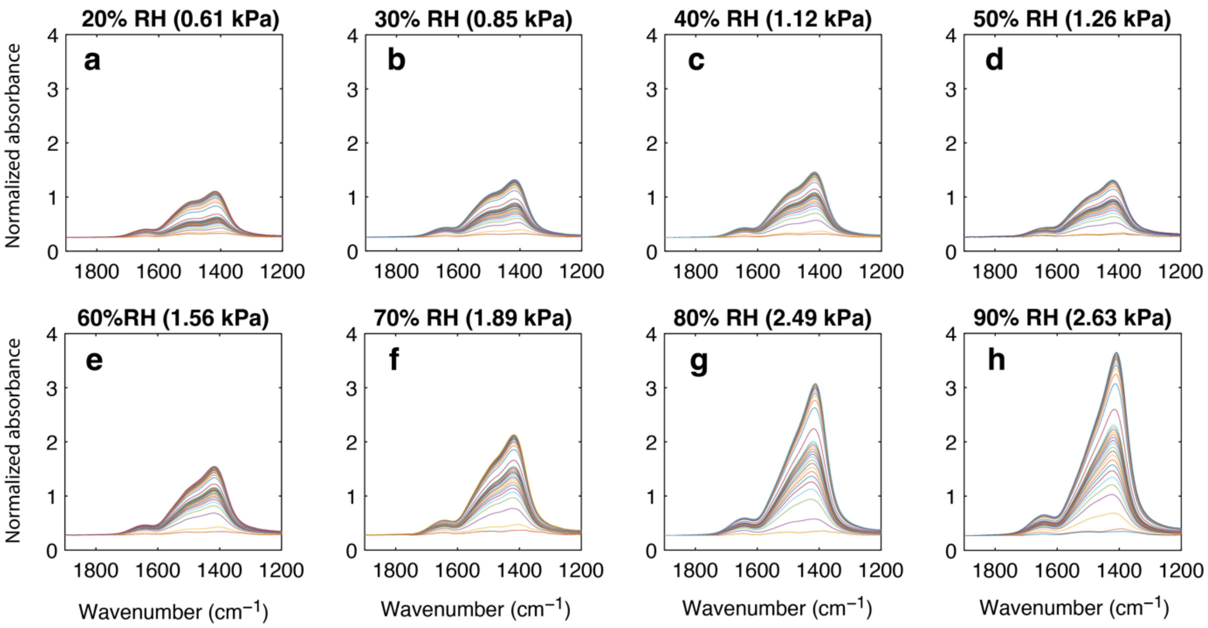

Figure S5. C-O stretching region of Pe5 particles exposed to a 507.6 mL/min flow of N_2(g) with (a-h) 0.61-2.63 kPa H_2O (g) (*i.e.* 20-90 % RH at 25 °C) and 2.6 kPa CO_2 for 20 h.

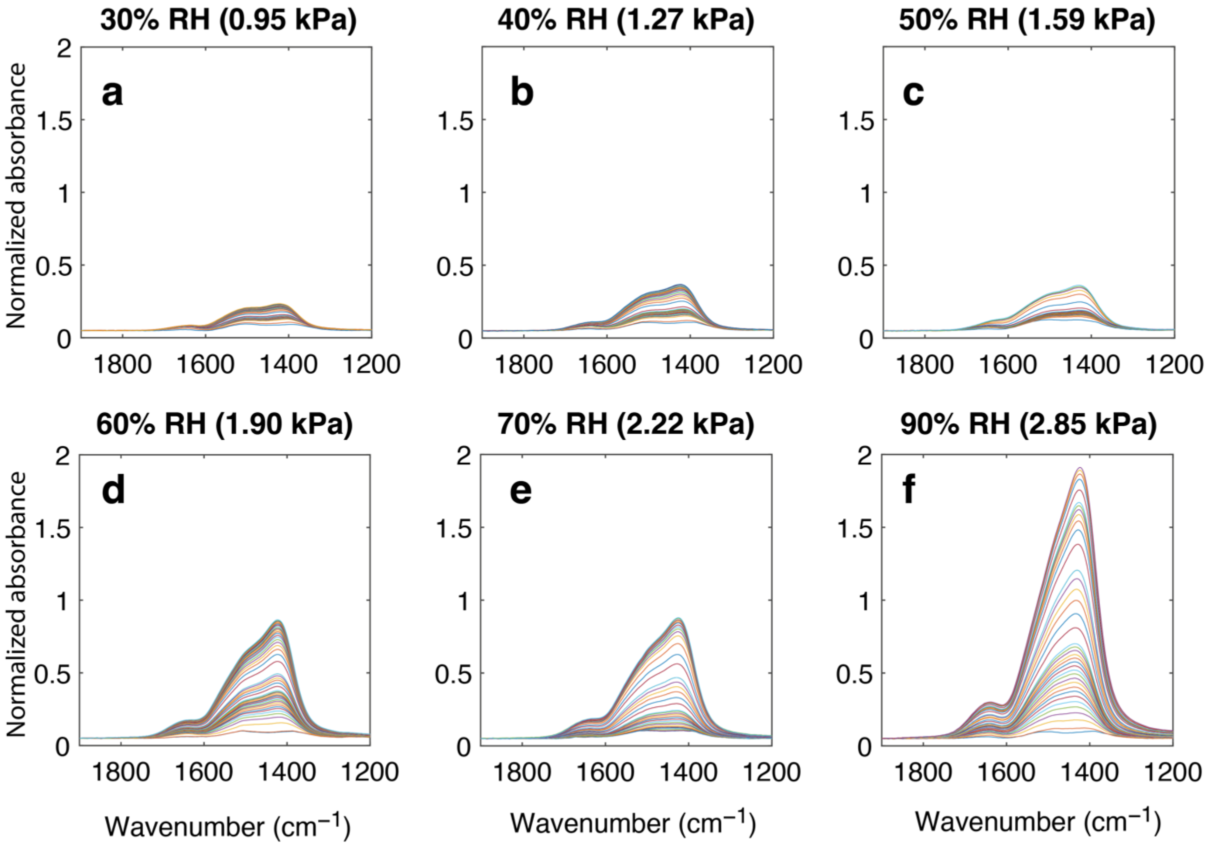

Figure S6. C-O stretching region of Pe10 particles exposed to a 507.6 mL/min flow of N_2(g) with (a-f) 0.95-2.85 kPa H_2O (g) (*i.e.* 30-90 % RH at 25 °C) and 2.6 kPa CO_2 for 20 h.

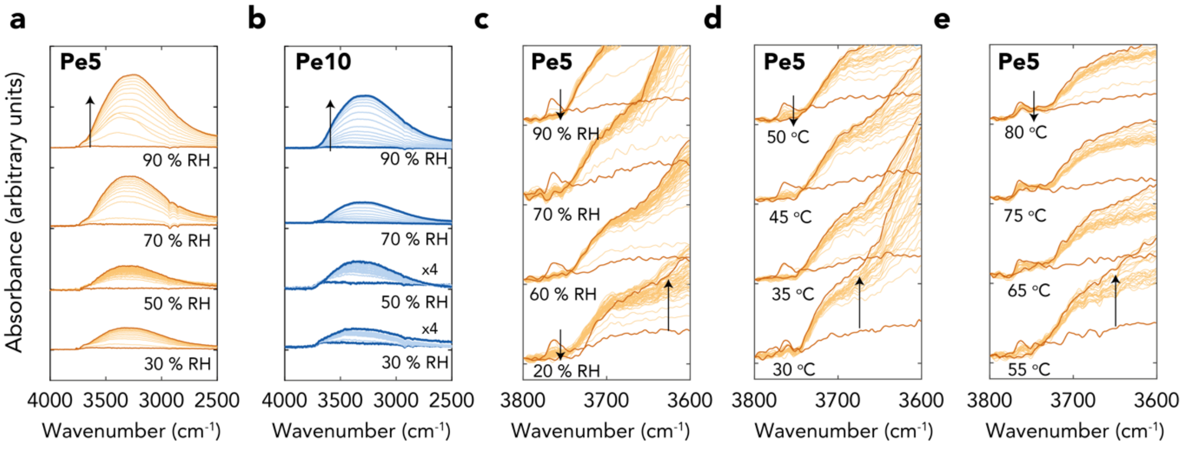

Figure S7. FTIR spectra of OH stretching regions of (a-c) humidity- and (d,e) temperature-dependent carbonation on (a,c-e) Pe5 and (b) Pe10 over 20-h period. Broad (a-b) and narrow (c) OH stretches show that water films grew but no discrete OH band of brucite (3701 cm^{-1}) appeared during carbonation. Additionally, surface OH group (3765 cm^{-1}) of periclase were more resilient on carbonation at high temperature (d,e), while they were rapidly consumed at room temperature at all humidity ranges (c). Arrows indicate the growth (↑) and disappearance (↓) of OH-bearing species.

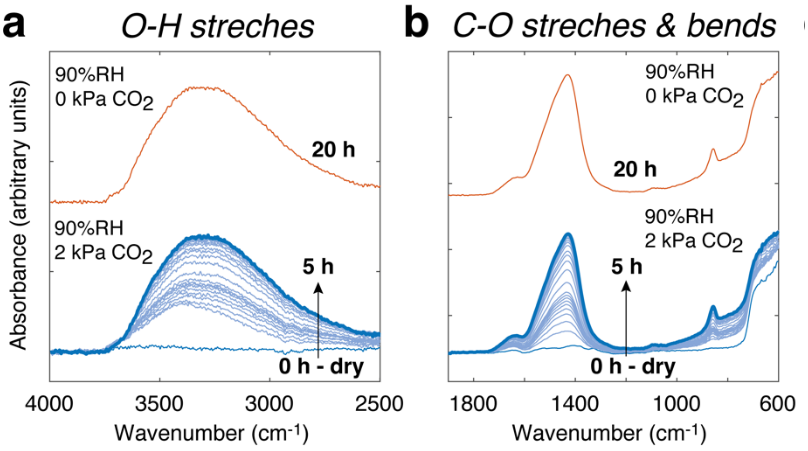

Figure S8. FTIR spectra of (a) OH stretching and (b) CO stretching and bending vibrations developed on periclase Pe5 exposed to 90% RH (N_2) and 2 → 0 kPa CO_2

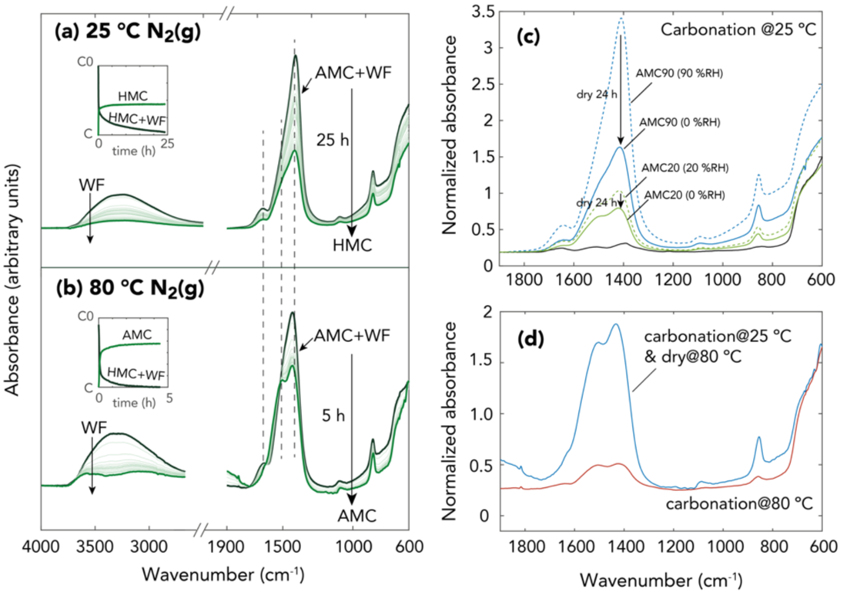

Figure S9. (a, b) Dehydration experiments of Pe5 first reacted under a flow of moist (90 % RH) 2kPa CO_2(g) in N_2(g) at 25 °C for 20 h, then exposed to dry N_2(g) at (a) 25 °C for 25 h and (b) 80 °C for 5 h. Dehydration removed free water molecules decrease intensity of C-O ν_3 singlet band (1414 cm^{-1}), leaving AMC signals (doublet C-O) with residual bulk water. Insets are concentration profiles obtained by a chemometric analysis[23] of these time-resolved spectra, showing the replacement a spectral component composed of AMC covered in a water film (AMC+WF) to a dry AMC. (c) Comparison of ν_3 band intensities of AMC+WF and AMC on carbonated Pe5 under 90% RH (blue) and 20% RH (green) at 25 ^{o}C. The spectra show less conversion at low humidity. (d) Comparison of ν_3 band intensities of 80 ^{o}C-dried AMC produced on Pe5 by carbonation at 90%RH at 25 ^{o}C (blue, this is also the last spectrum in (b)) and 80 ^{o}C (red). The spectra show less conversion at high temperature.

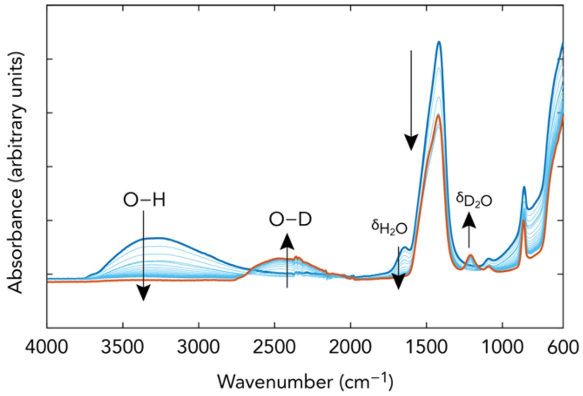

Figure S10. Deuteration of reacted periclase Pe5 (under 90%RH H_2O and 2.6 kPa CO_2 at 28 °C for 20 h). After exposure to humid CO_2 atmosphere, the reacted periclase was switched to a flow of N_2(g)/D_2O mixture (D_2O vapor partial pressure equivalent to ~13% RH). This condition created a sub-monolayer heavy water films on AMC.

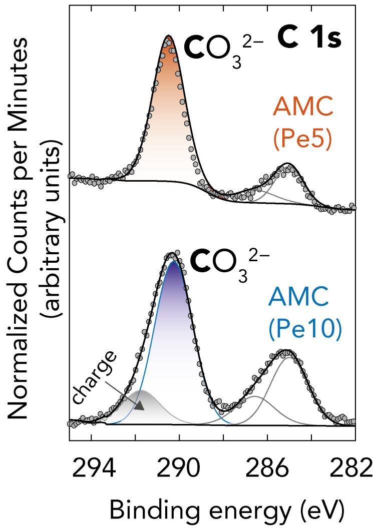

Figure S11. Example of XPS in the C1s region of Pe5 and Pe10 samples reacted in 2.6 kPa CO_2 with 90 % RH for 20 h.

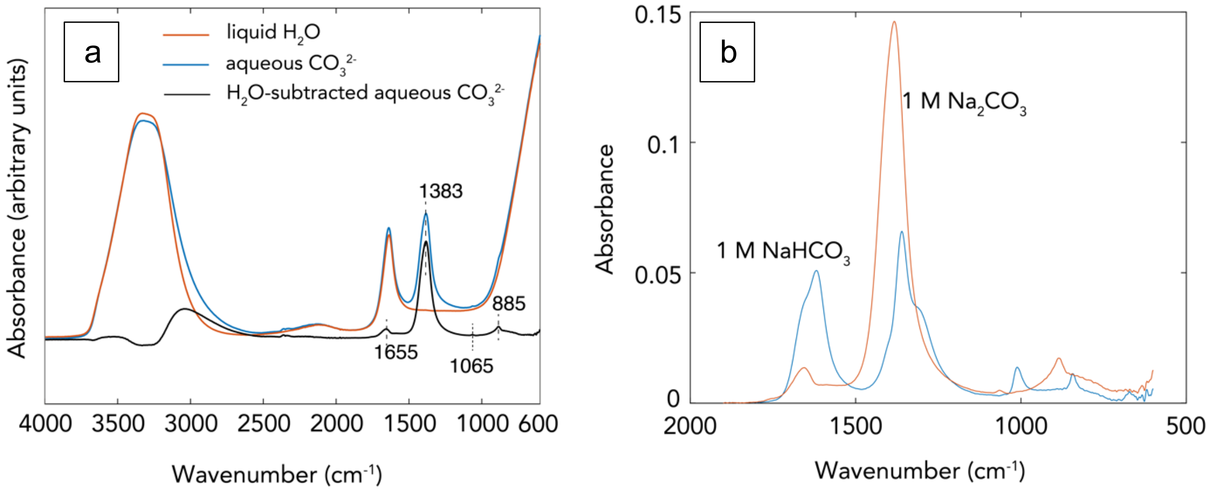

Figure S12. (a) ATR-FTIR spectra of aqueous CO_3^{2-} (1 M Na_2CO_3, blue), liquid H_2O (red). H_2O-subtracted FTIR spectrum of aqueous CO_3^{2-} (black) shows the predominant degenerate C–O stretches of the hydrated carbonate (D_{3h} symmetry) ions at 1383 cm^{-1}. (b) Comparison of C-O stretches of free hydrated CO3 (red) and HCO3 (blue) ions of 1 M concentration. The absorbance of the two equal molar concentration species is thus directly related to molar absorptivity ($\varepsilon = A/lC$).

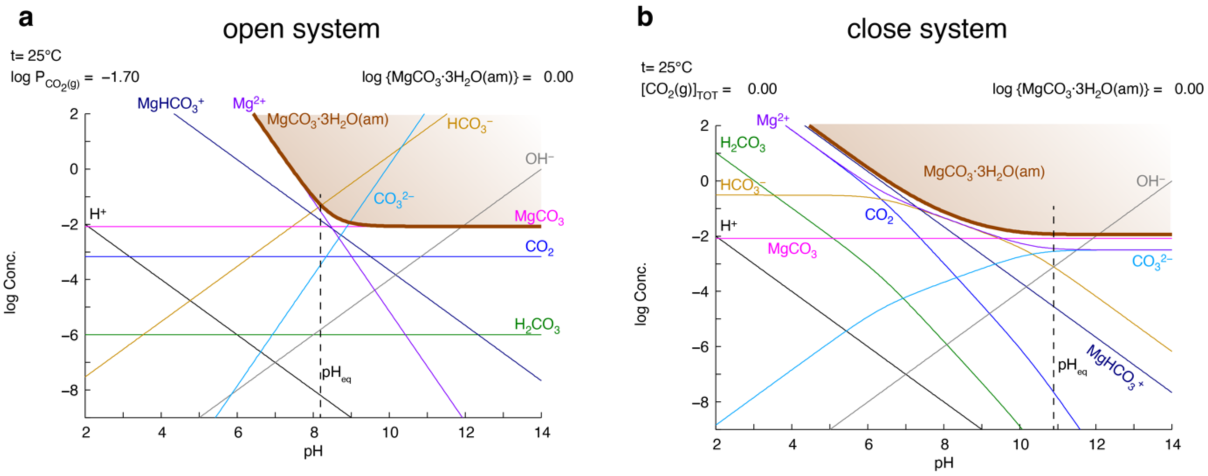

Figure S13. Chemical speciation diagram of $MgCO_3$-CO_2-H_2O system at 25 °C. (a) Open system that is constantly equilibrated with CO_2(g) (pCO_2(g) = $10^{-1.7}$ atm = ~ 2.6 kPa) showing free and ion pairs of (bi)carbonate are dominant species. The pH of the system is 8.15, calculated from the reduced charge balance condition 2[Mg^{2+}] = [HCO_3^-]. (b) Close system ([CO_2(g)]$_{TOT}$ = 0). In both cases, the activity of solid phase amorphous magnesium carbonate is 1 (log{$MgCO_3$}= 0). Diagrams were generated with the program SPANA/HYDRA/MEDUSA (https://github.com/ignasi-p/eq-diagr-kth) using the equilibrium constants of Table S3.

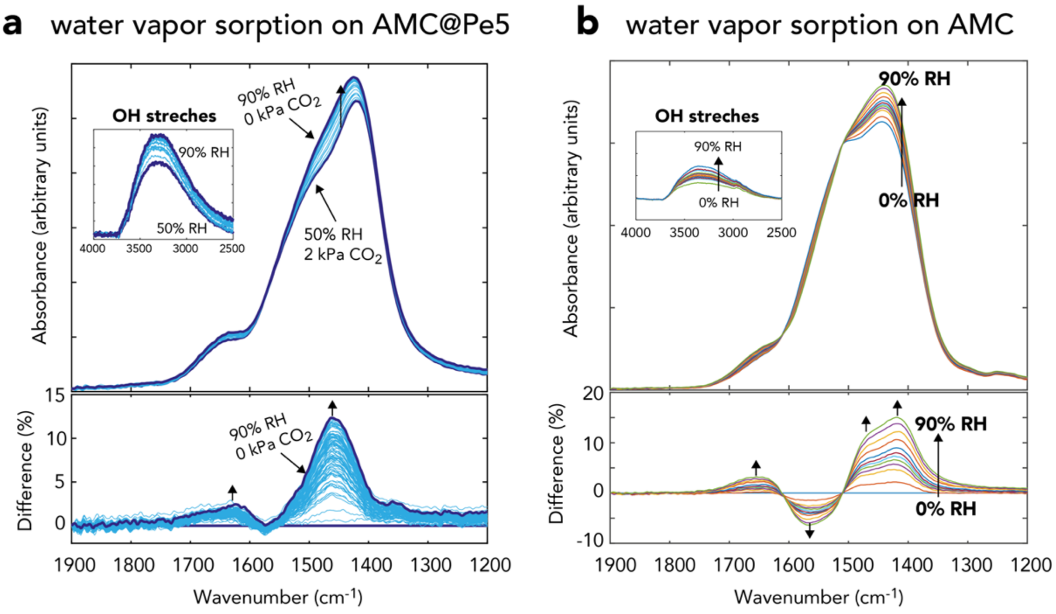

Figure S14. (a) Post-hydration of growth AMC from Pe5 under 2.6 kPa CO_2 and 50%RH. *Top*: raw C-O stretches of AMC appeared as doublet by the reaction but became a singlet under CO_2-free humid atmosphere of 90% RH. *Inset*: OH stretching region. *Bottom*: difference spectra of the C-O stretches show the rise of singlet ~1450 cm^{-1} (b) Water vapor adsorption experiment on synthetic AMC was conducted under CO_2-free atmosphere with varied humidity from 0-90% RH. *Top*: raw C-O stretches changes their shape and intensity upon hydration. *Inset*: OH streching region. *Bottom*: difference spectra of the C-O stretches.

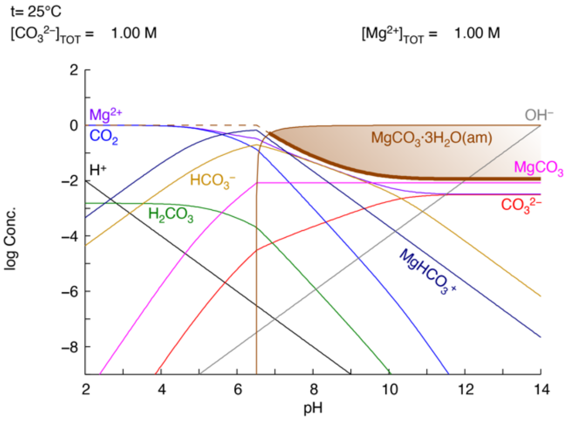

Figure S15. Chemical speciation diagram of the Mg^{2+}-CO_3^{2-}-H_2O system at 25 °C. Diagrams were generated with the program SPANA/HYDRA/MEDUSA (https://github.com/ignasi-p/eq-diagr-kth) using the equilibrium constants of Table S4. The system is generally similar to the diagram in Fig S13b in the pH > 8 region, where solid carbonate is in equilibrium with water. The equilibrium pH in this case is also determined at pH 10.8.

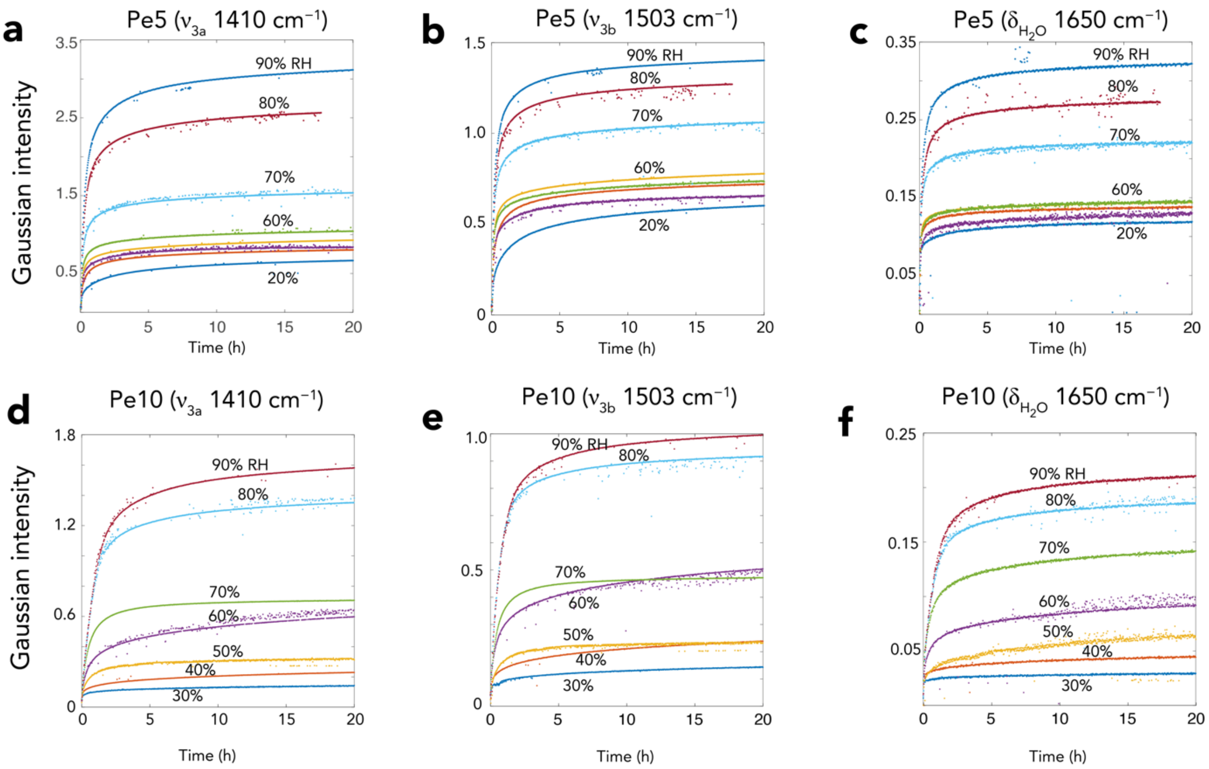

Figure S16. Time-resolved Gaussian component band intensities of the C-O stretching region of carbonate (a,b,d,e) and of the water bending region (c,f) in Pe5 (a-c) and Pe10 (d-f) during reactions with 2.6 kPa CO_2 and 90% RH at 25 °C.

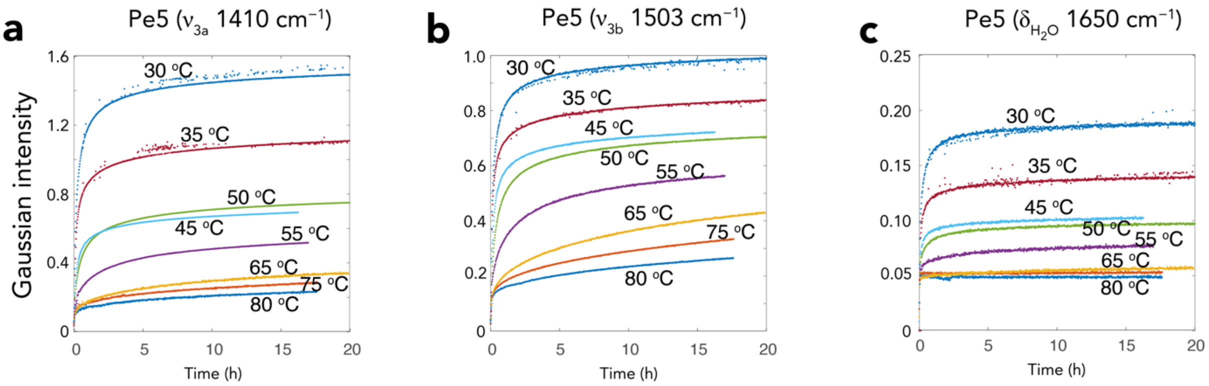

Figure S17. Time-resolved Gaussian component band intensities of the C-O stretching region of carbonate (a,b) and of the water bending region (c) in Pe5 during reactions 2.6 kPa CO_2 with 90% RH at 30-80 °C.

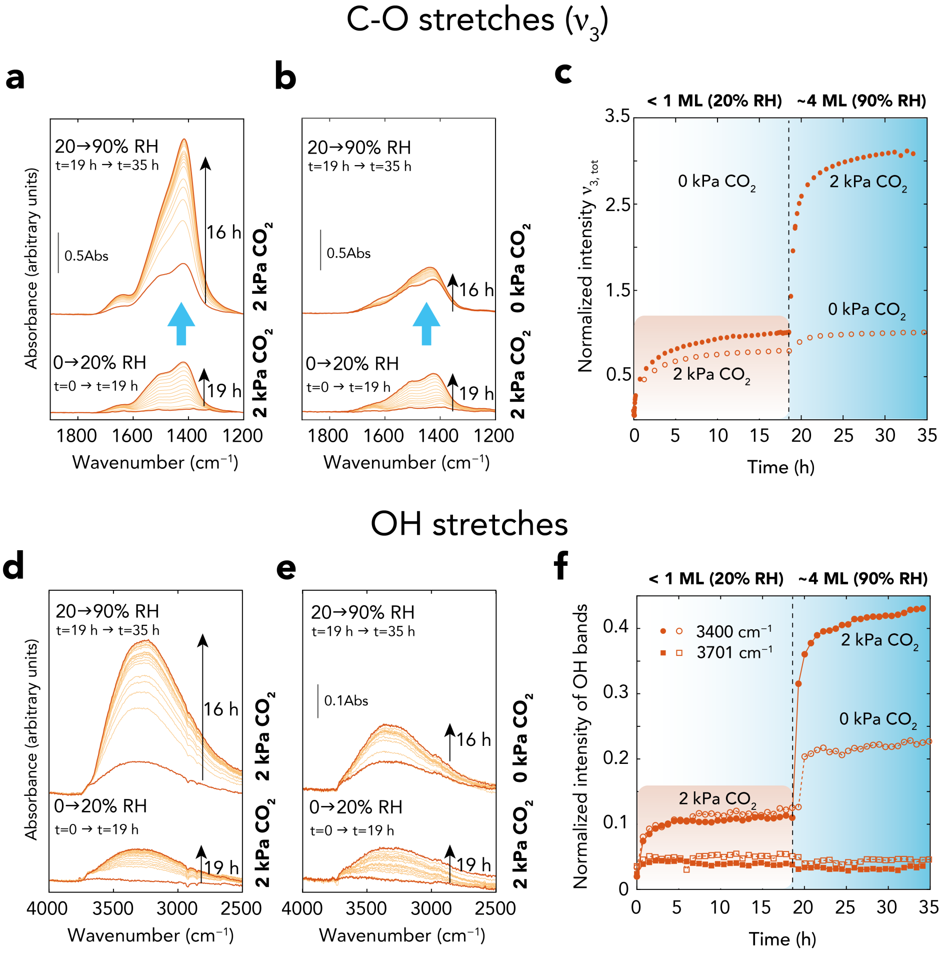

Figure S18. Solubility test on the AMC nanocoatings. FTIR spectra of time-resolved growth of the CO stretches ν_{3tot} (a, b) and OH stretches (d, e) and their corresponding band intensities (c, f) of periclase Pe5 reacted first under a stream of 20% RH and 2.6 kPa CO_2 for 19 h (bottom spectra), followed by continue reaction under a stream of 90% RH with 2.6 kPa CO_2 or 0 kPa CO_2 over the next 16 h (top spectra). Intensity profiles of (c) CO stretches show continuous production of AMC from pre-grown AMC under CO_2-rich 4 ML water films, while exposing this MgO@AMC to CO_2-free 4 ML water films only promote hydration effect on carbonate species of AMC. Intensity profiles of (f) OH stretching bands show no appearance of distinct OH group belong to brucite (3701 cm^{-1}) under both reaction conditions, while OH bands of water films (3400) grew thicker under CO_2-rich 4 ML water films.

TABLES

TABLE S1 **X-ray photoelectron spectroscopy of Pe5**

	Pe5		Pe5-CO2-30RH		Pe5-CO2-50RH		Pe5-CO2-90RH	
	BE (FWHM), eV	at.%	BE (FWHM), eV	at.%	BE (FWHM), eV	at.%	BE (FWHM), eV	at.%
C 1s	285.0 (1.95)	1.69	285.6 (1.70)	3.93	285.0 (1.50)	11.44	285.0 (1.45)	2.19
			287.2 (1.90)	1.22	286.3 (1.80)	3.14	286.6 (2.10)	1.08
					288.5 (2.30)	1.52		0.98
C 1s CO_3^{2-}	289.9 (1.85)	2.17	290.7 (1.80)	7.84	290.7 (1.90)	8.98	290.5 (1.70)	9.70
C 1s HCO_3^{-}								
O 1s O	529.4 (1.50)	38.52	530.0 (1.50)	15.46	529.8 (1.50)	12.52	529.8 (1.45)	5.47
O 1s OH	531.5 (1.85)	21.43						
O 1s CO_3^{2-}	532.8 (1.65)	1.74	532.4 (2.30)	42.35	532.2 (2.40)	45.61	532.2 (1.80)	51.03
O 1s COO			534.4 (1.80)	2.27			534.1 (2.00)	5.00
Mg 1s	1302.4 (1.55)	26.88	1303.0 (1.60)	4.53	1302.7 (1.70)	4.73	1302.5 (1.95)	1.12
	1303.7 (1.90)	7.57	1304.6 (2.40)	16.79	1304.3 (2.10)	12.06	1304.4 (1.85)	19.44
F 1s							685.5 (2.10)	4.98

TABLE S2 **X-ray photoelectron spectroscopy of Pe10**

	Pe10-CO2-90RH	
	BE (FWHM), eV	at.%
C 1s	285.0 (1.95)	4.87
	286.6 (2.10)	2.22
C 1s CO_3^{2-}	290.3 (1.95)	11.77
C 1s HCO_3^{-}	291.8 (2.00)	2.49*
O 1s O	529.6 (1.35)	8.29
O 1s OH		
O 1s CO_3^{2-}	531.8 (1.90)	45.65
O 1s COO	533.3 (1.95)	8.00
Mg 1s	1302.5 (1.55)	0.96
	1304.1 (2.00)	15.75
F 1s		

* charging issue, not a signal of bicarbonate specie.

TABLE S3 **Thermodynamic constants for $MgCO_3$-CO_2-H_2O system***

	H^+	$CO_2(g)$	$MgCO_3.3H_2O$ (am)	Log K
H^+	1			
OH^-	–1			–14
HCO_3^-	–1	1	0	–7.825
CO_3^{2-}	–2	1	0	–18.152
Mg^{2+}	2	–1	1	13.152
$MgHCO_3^+$	1	0	1	6.34
$MgCO_3^0$	0	0	1	–2.08
H_2CO_3*	0	1	0	–4.293
$CO_2(aq)$	0	1	0	–1.472
$MgCO_3.3H_2O$ (am)			1	

*All reaction constants (log*K*) were collectively derived from these references: NIST database[17] from SPANA's default database, work of Plummer and co-workers,[24] and work of Wang and co-wokers[25]. Solubility product of AMC ($MgCO_3.3H_2O$ (am)) was taken as an average (log*K* = - 5) from values in literatures[14-16].

TABLE S4 **Thermodynamics constants for Mg^{2+}-CO_3^{2-}-H_2O system (closed system)**

	H^+	CO_3^{2-}	Mg^{2+}	log K
H^+	1			
OH^-	–1			–14
$CO_2(aq)$	2	1	0	16.68
H_2CO_3	2	1	0	13.859
HCO_3^-	1	1	0	10.327
$MgHCO_3^+$	1	1	1	11.34
$MgCO_3^0$	0	1	1	2.92
$CO_2(g)$	2	1	0	18.152
$MgCO_3.3H_2O$ (am)	0	1	1	5.0
Mg^{2+}	0	0	1	
CO_3^{2-}	0	1	0	

*All reaction constants (log*K*) were collectively derived from these references: NIST database[17] from SPANA's default database, work of Plummer and co-workers,[24] and work of Wang and co-wokers[25]. Solubility product of AMC ($MgCO_3.3H_2O$ (am)) was taken as an average (log*K* = - 5) from values in literatures[14-16].

REFERENCES

(1) Eubank, W. R. Calcination Studies of Magnesium Oxides. *J. Am. Ceram. Soc.* **1951**, *34* (8), 225-229.
(2) Phillips, V. A.; Opperhauser, H.; Kolbe, J. L. Relations Among Particle Size, Shape, and Surface Area of Mg(OH)2 and Its Calcination Product. *J. Am. Ceram. Soc.* **1978**, *61* (1-2), 75-81.
(3) Green, J. Calcination of precipitated $Mg(OH)_2$ to active MgO in the production of refractory and chemical grade MgO. *J. Mat. Sci.* **1983**, *18* (3), 637-651.
(4) Layden, G. K.; Brindley, G. W. Kinetics of Vapor-Phase Hydration of Magnesium Oxide. *Journal of the American Ceramic Society* **1963**, *46* (11), 518-522.
(5) Feitknecht, W.; Braun, H. Der Mechanismus der Hydratation von Magnesiumoxid mit Wasserdampf. *Helv. Chim. Acta* **1967**, *50* (7), 2040-2053.
(6) Smithson, G. L.; Bakhshi, N. N. The kinetics and mechanism of the hydration of magnesium oxide in a batch reactor. *Can. J. Chem. Eng.* **1969**, *47* (6), 508-513.
(7) Martin, M.; Jindřich, B. Hydration Kinetics of Magnesium Oxide - Part 3. Hydration Rate of MgO in Terms of Temperature and Time of Its Firing. *Ceramics-Silikáty* **1997**, *41* (4), 121-123.
(8) Birchal, V. S. S.; Rocha, S. D. F.; Ciminelli, V. S. T. The effect of magnesite calcination conditions on magnesia hydration. *Minerals Eng.* **2000**, *13* (14), 1629-1633.
(9) Salomão, R.; Arruda, C. C.; Kawamura, M. A. A systemic investigation on the hydroxylation behavior of caustic magnesia and magnesia sinter. *Ceram. Int.* **2015**, *41* (10, Part B), 13998-14007.
(10) Baumann, S. O.; Schneider, J.; Sternig, A.; Thomele, D.; Stankic, S.; Berger, T.; Grönbeck, H.; Diwald, O. Size Effects in MgO Cube Dissolution. *Langmuir* **2015**, *31* (9), 2770-2776.
(11) Anderson, P. J.; Horlock, R. F. Thermal decomposition of magnesium hydroxide. *Transactions of the Faraday Society* **1962**, *58*, 1993-2004.
(12) Goodman, J. F. The decomposition of magnesium hydroxide in an electron microscope. *Prof. Royal Soc. London.* **1958**, *247* (1250), 346-&.
(13) Kim, M. G.; Dahmen, U.; Searcy, A. W. Structure transformations in the decomposition of Mg(OH)2 and MgCO3. *J. Am. Ceramics Soc.* **1987**, *70* (3), 146-154.
(14) Purgstaller, B.; Goetschl, K. E.; Mavromatis, V.; Dietzel, M. Solubility investigations in the amorphous calcium magnesium carbonate system. *CrystEngComm* **2019**, *21* (1), 155-164.
(15) Mergelsberg, S. T.; De Yoreo, J. J.; Miller, Q. R. S.; Marc Michel, F.; Ulrich, R. N.; Dove, P. M. Metastable solubility and local structure of amorphous calcium carbonate (ACC). *Geochim. Cosmochim. Acta* **2020**, *289*, 196-206.
(16) Chang, C.-Y.; Yang, S.-Y.; Chan, J. C. C. Solubility product of amorphous magnesium carbonate. *J. Chinese Chem. Soc.* **2021**, *68* (3), 476-481.
(17) NIST Standard Reference Database 46 Version 8. Institute of Standards and Technology, . http\://www.nist.gov/srd/nist46.cfm\nNational (accessed.
(18) Downs, R. T.; Hall-Wallace, M. The American Mineralogist crystal structure database. *Am. Mineral.* **2003**, *88* (1), 247-250.
(19) Hazen, R. M. Effects of temperature and pressure on the cell dimension and X-ray temperature factors of periclase. *Am. Mineral.* **1976**, *61* (3-4), 266-271.
(20) Catti, M.; Ferraris, G.; Hull, S.; Pavese, A. Static compression and H disorder in brucite, Mg(OH)2, to 11 GPa: a powder neutron diffraction study. *Phys. Chem. Min.* **1995**, *22* (3), 200-206.
(21) Thomele, D.; Baumann, S. O.; Schneider, J.; Sternig, A. K.; Shulda, S.; Richards, R. M.; Schwab, T.; Zickler, G. A.; Bourret, G. R.; Diwald, O. Cubes to Cubes: Organization of MgO Particles into One-Dimensional and Two-Dimensional Nanostructures. *Crystal Growth Des.* **2021**, *21* (8), 4674-4682.
(22) Thomele, D.; Bourret, G. R.; Bernardi, J.; Bockstedte, M.; Diwald, O. Hydroxylation Induced Alignment of Metal Oxide Nanocubes. *Angew Chem Int Ed Engl* **2017**, *56* (5), 1407-1410.
(23) Jaumot, J.; Gargallo, R.; de Juan, A.; Tauler, R. A graphical user-friendly interface for MCR-ALS: a new tool for multivariate curve resolution in MATLAB. *Chem. Intel. lab. Sys.* **2005**, *76* (1), 101-110.
(24) Plummer, L. N.; Busenberg, E. The solubilities of calcite, aragonite and vaterite in CO_2-H_2O solutions between 0 and 90°C, and an evaluation of the aqueous model for the system $CaCO_3$-CO_2-H_2O. *Geochim. Cosmochim. Acta* **1982**, *46* (6), 1011-1040.
(25) Wang, X.; Conway, W.; Burns, R.; McCann, N.; Maeder, M. Comprehensive Study of the Hydration and Dehydration Reactions of Carbon Dioxide in Aqueous Solution. *J. Phys. Chem. A* **2010**, *114* (4), 1734-1740.
